# Supplementary material for: Combined MYC and P53 Defects Emerge at Medulloblastoma Relapse and Define Rapidly Progressive, Therapeutically Targetable Disease
Source: Cancer Cell. 2015 Jan 12;27(1):72–84. doi: 10.1016/j.ccell.2014.11.002 (PMC4297293; doi:10.1016/j.ccell.2014.11.002)
Supplement: Document S1. Supplemental Experimental Procedures, Figures S1–S4, and Tables S1–S5 [file mmc1.pdf]

Cancer Cell, Volume *27*

Supplemental Information

Combined MYC and P53 Defects Emerge at Medulloblastoma Relapse and Define Rapidly Progressive, Therapeutically Targetable Disease

Rebecca M. Hill, Sanne Kuijper, Janet C. Lindsey, Kevin Petrie, Ed C. Schwalbe, Karen Barker, Jessica K.R. Boult, Daniel Williamson, Zai Ahmad, Albert Hallsworth, Sarra L. Ryan, Evon Poon, Simon P. Robinson, Ruth Ruddle, Florence I. Raynaud, Louise Howell, Colin Kwok, Abhijit Joshi, Sarah Leigh Nicholson, Stephen Crosier, David W. Ellison, Stephen B. Wharton, Keith Robson, Antony Michalski, Darren Hargrave, Thomas S. Jacques, Barry Pizer, Simon Bailey, Fredrik J. Swartling, William A. Weiss, Louis Chesler, and Steven C. Clifford

SUPPLEMENTAL DATA

Table S1 (related to Table1). Molecular details of *CTNNB1* and *TP53* mutated medulloblastomas

Patient number	Mutated gene	Protein	p53 database[1]	Acquired mutation	Molecular subgroup
13	*CTNNB1*	Ser34Phe	n/a	No	MB_{WNT}
14	*CTNNB1*	Ser37deletion	n/a	Unknown	MB_{WNT}
5	*TP53*	Gly245Val	Yes	Yes	MB_{SHH}
8	*TP53*	Arg282Trp	Yes	No	MB_{SHH}
10	*TP53*	Arg282Trp	Yes	No	MB_{SHH}
12	*TP53*	Arg273His	Yes	No	MB_{SHH}
13	*TP53*	Arg158Cys and Arg282Trp	Yes	No	MB_{WNT}
14	*TP53*	Arg273His	Yes	Unknown	MB_{WNT}
22	*TP53*	Pro152Leu	Yes	Yes	MB_{Group4}
29	*TP53*	Arg175His	Yes	Unknown	Unknown

[1](Soussi et al. 2006) Ser, Serine; Phe, phenylalanine; Arg, Arginine; Cys, Cysteine; Trp, Tryptophan; His, Histidine; Gly, Glycine; Val, Valine; Pro, Proline; Leu, Leucine. n/a, not applicable. *CTNNB1* encodes Beta-catenin.

Table S2 (related to Table1). Subgroup distribution in the present paired study cohort of relapsed medulloblastomas compared to previously published data

Independent published cohort of medulloblastomas sampled at relapse[1] (Ramaswamy et al., 2013)

Molecular subgroup	Paired relapse study	Independent published study	
	Relapse	Relapse	p value
MB_{SHH}	12/25 (48%)	21/51 (41%)	0.6275
MB_{WNT}	2/25 (8%)	1/51 (2%)	0.2504
MB_{Group3}	2/25 (8%)	9/51 (18%)	0.3215
MB_{Group4}	9/25 (36%)	20/51 (39%)	1

Relapsing tumours in an unbiased trial-based cohort of medulloblastomas sampled at diagnosis[2] (Schwalbe et al., 2013)

Molecular subgroup	Paired relapse study	Independently published study	
	Relapse	Relapsing tumours (sampled at dignosis)	p value
MB_{SHH}	5/18 (28%)	17/64 (27%)	1
MB_{WNT}	2/18 (11%)	1/64 (2%)	0.1198
MB_{Group3}	2/18 (11%)	16/64 (25%)	0.3349
MB_{Group4}	9/18 (50%)	30/64 (47%)	1

[1]Based on all patients with available subgroup data.
[2]Based on all patients receiving radiotherapy aged 3-16 years. p, Fisher's exact test.

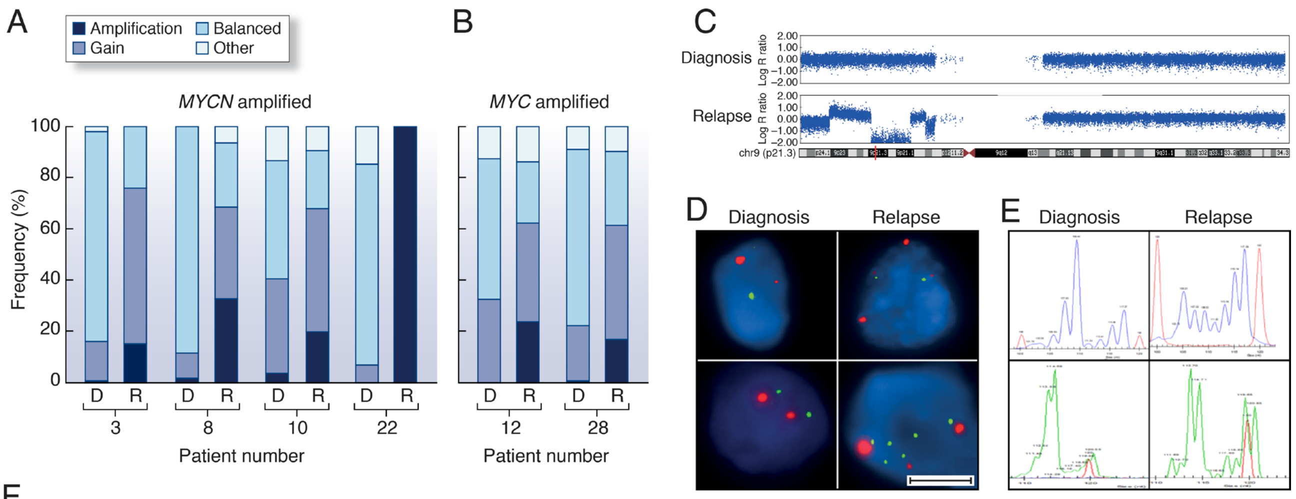

F

Treatment group	Overall survival
Non-infant: RT at diagnosis	0/22 (0%)[1]
Non-infant: RT at relapse	0/1 (0%)
Infant: No RT	0/2 (0%)
Infant: RT at relapse	4/4 (100%)

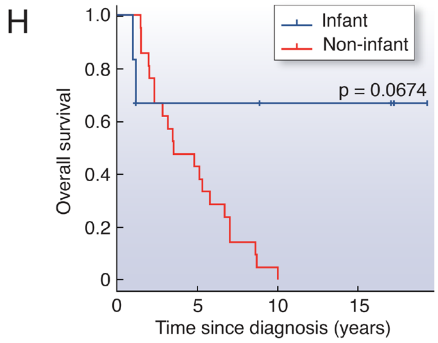

G

Variable		Number of cases n = 29	Univariate			Multivariate
			**HR**	**95% CI**	**p value**	**p value**
Age	Infant	6/29 (21%)	0.171	0.04 – 0.77	**0.021**	**0.021**
	Non-infant	23/29 (79%)				
Gender	Male	18/29 (62%)	0.385	0.16 – 0.92	**0.032**	ns
	Female	11/29 (38%)				
RT at diagnosis	Yes	22/29 (76%)	3.715	1.05 – 13.21	**0.043**	ns
	No	7/29 (24%)				
Resection	STR	9/29 (31%)	2.281	0.9 – 5.79	0.083	ns
	GTR	20/29 (69%)				
Pathology	LCA	3/26 (12%)	1.758	0.49 – 6.33	0.388	ns
	Non-LCA	23/26 (88%)				
Metastatic stage	M+	10/29 (34%)	0.886	0.37 – 2.10	0.784	ns
	M–	19/29 (66%)				

Figure S1 (related to Figure 1). Analysis of enrichment and acquisition of molecular defects at relapse and patient survival following medulloblastoma recurrence

(A) Frequency as determined by iFISH of intra-tumoral *MYCN* copy number defects at diagnosis (D) and relapse (R).

(B) Frequency as determined by iFISH of intra-tumoral *MYC* copy number defects at diagnosis (D) and relapse (R).

(C) *CDKN2A* (*p14*^{ARF}) homozygous deletion at relapse (Illumina Omni-express SNP array; *CDKN2A* position, red).

(D) Evolution of chromosome 17 defects and polyploidy by iFISH. (top left) 17p loss (green versus centromeric control (red)), (bottom left) balanced 17q, (top right) maintenance of 17p loss with development of polyploidy, (bottom right) acquisition of polyploidy and 17q gain (overall i(17q)). Scale bar indicates 5 μM.

(E) Acquisition of microsatellite instability at relapse (diagnosis; normal heterozygous profiles and relapse; multiple peaks demonstrating instability).

(F) Survival status of 29 relapsed medulloblastoma patients by treatment received. Infant, < 4.0 years at diagnosis; RT, radiotherapy. [1]Two patients died from treatment complications.

(G) Unadjusted Cox proportional hazard models assessing the prognostic significance (overall survival) of clinico-pathological variables at diagnosis in relapsing patients. STR, subtotal resection; GTR, gross total resection; LCA, large-cell/anaplastic; M+, M2+ disease; M-, M0/M1.

(H) Kaplan-Meier curve (whole cohort) showing survival of infant patients following recurrence (p, log-rank test, Bonferroni corrected).

Table S3 (related to Figure 1). Incidence of clinical, pathological and molecular features in the present relapsed study cohort (at diagnosis and relapse) compared to large historic cohorts

Clinicopathological and molecular features		Historic studies[1]	Paired relapse study			
		**Diagnosis**	**Diagnosis**	**p value**	**Relapse**	**p value**
Gender	Male	585/952 (61%)	18/29 (62%)		18/29 (62%)	
	Female	367/952 (39%)	11/29 (38%)	1	11/29 (38%)	1
	Male:female ratio	1.6:1	1.6:1		1.6:1	
Age	Age range in years	0.3–52	0.1–33.7			
	Infants (<4 years)	167/943 (17%)	6/29 (21%)	0.6261*		
	Children (4–16 years)	599/943 (64%)	22/29 (76%)			
	Adults (>16 years)	177/943 (19%)	1/29 (3%)			
Pathology	CLA	938/1277 (74%)	16/26 (61%)		14/24 (58%)	
	DN	183/1277 (14%)	7/26 (27%)		4/24 (17%)	
	LCA	156/1277 (12%)	3/26 (12%)	1*	6/24 (25%)	0.1073*
Metastatic stage	M–	608/834 (73%)	9/22 (41%)	**0.0026**	6/24 (25%)	**<0.0001**
	M+	226/834 (27%)	13/22 (59%)		18/24 (75%)	
Resection	GTR	267/317 (84%)	20/29 (69%)	0.0657	n/a	
	STR	50/317 (16%)	9/29 (31%)		n/a	
Molecular subgroup	MB_{SHH}	153/550 (28%)	12/25 (48%)	**0.0402***	12/25 (48%)	**0.0402***
	MB_{WNT}	60/550 (11%)	2/25 (8%)	1	2/25 (8%)	1
	MB_{Group3}	149/550 (27%)	2/25 (8%)	**0.0355***	2/25 (8%)	**0.0355***
	MB_{Group4}	188/550 (34%)	9/25 (36%)	0.8324	9/25 (36%)	0.8324
Molecular defects	*MYC/MYCN* amplification	50/552 (9%)	2/25 (8%)	1	9/29 (31%)	**0.0012**
	MYC amplification	17/552 (3%)	1/25 (4%)	0.5549	4/29 (14%)	**0.0166**
	MYCN amplification	33/552 (6%)	1/25 (4%)	1	5/29 (17%)	**0.034**
	TP53 mutations	21/310 (7%)	4/26 (15%)	0.1157	8/29 (28%)	**0.0013**
	Chromosome 17 defects	138/260 (53%)	8/21 (38%)	0.2562	10/21 (48%)	0.6562
Combined molecular defects	*TP53* mutation and *MYC/MYCN* amplification	8/310 (3%)	0/26 (0%)	1	6/29 (21%)	**0.0004**
	TP53 mutation and *MYC* amplification	1/310 (<1%)	0/26 (0%)	1	2/29 (7%)	**0.0201**
	TP53 mutation and *MYCN* amplification	7/310 (2%)	0/26 (0%)	1	4/29 (14%)	**0.0094**

[1]Historic studies: (Kool et al,. 2012; Pfister et al,. 2009; Ryan et al,. 2012; McManamy et al,. 2007; Lannering et al,. 2012; Pfaff et al,. 2010). Pathology variant: CLA, classic; LCA, large-cell/anaplastic; DN, desmoplastic/nodular. Metastatic stage: M-, M0; M+, M1-4. Resection: GTR, gross total resection; STR sub-total resection. Data are shown as a proportion and percentage of all data available. *Patients with each specific feature compared against all others. Features significantly enriched at diagnosis and relapse are in bold (p, Fisher's exact test). n/a, not applicable.

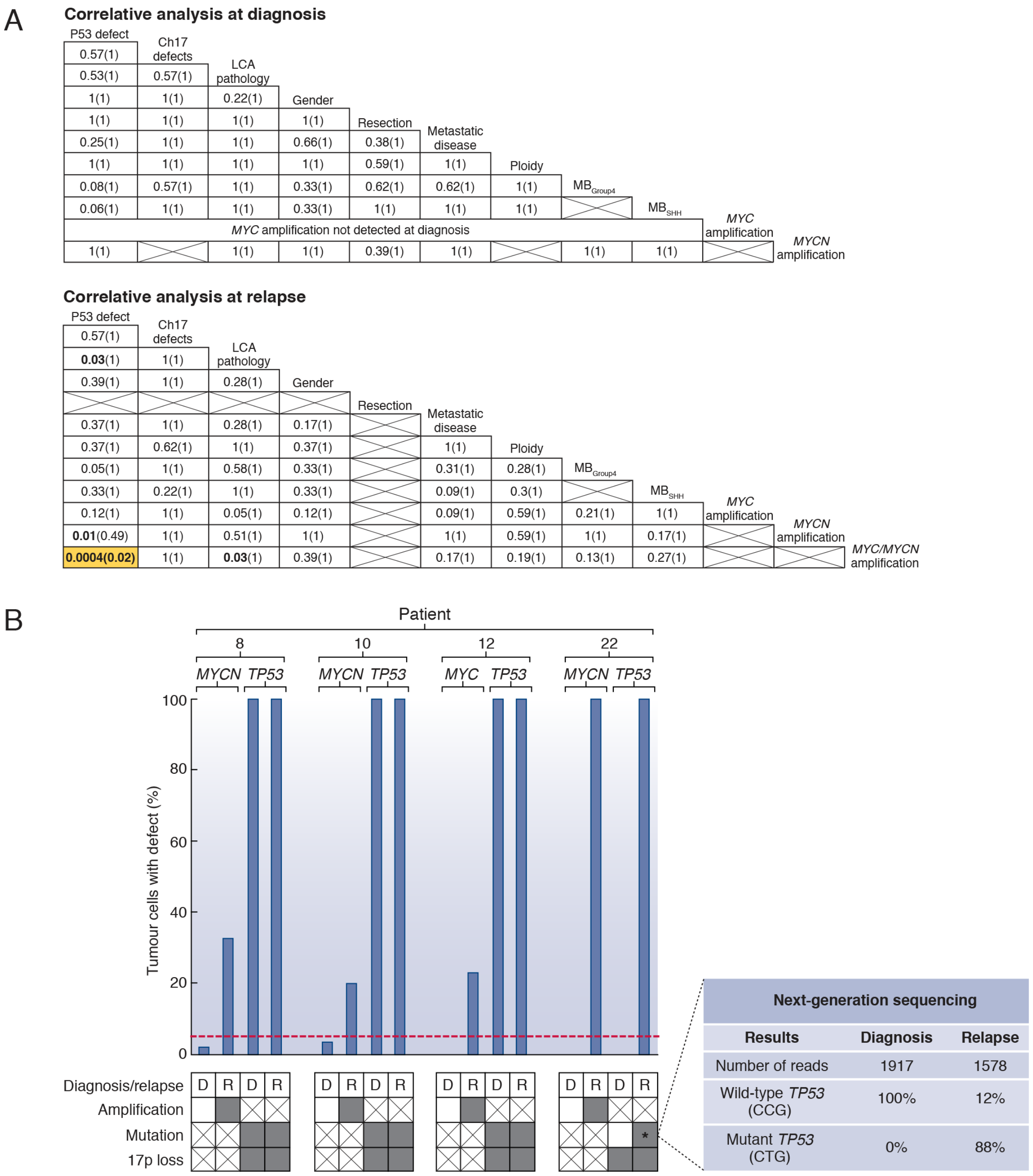

Figure S2 (related to Figure 2). Analysis of combined P53 pathway defects and *MYC/MYCN* amplification at relapse

(A) *MYC/MYCN* amplification and P53 pathway defects at relapse are significantly correlated. Correlative p values (p, Fisher's exact test) at diagnosis and relapse are shown for patients receiving standard upfront radiotherapy and chemotherapy before and after (in parentheses) adjustment for multiple testing (Bonferroni correction). Cross-hatched boxes, not relevant.

(B) Assessment of intra-tumoral molecular heterogeneity supports the development of combined *MYC* family gene amplification and *TP53* mutation by both clonal enrichment and de novo acquisition. Estimated percentages of *MYC/MYCN*-amplified (by iFISH; >5% cells, dashed red line) and *TP53* mutated (estimated peak heights, mutant versus wild-type) tumor cells at diagnosis (D) and relapse (R) in four patients with available material support clonal enrichment (e.g. *MYCN* amplification in patients 8 & 10) and de novo acquisition of combined defects at relapse (e.g. *MYC* amplification in patient 12; *MYCN* amplification and *TP53* mutation in patient 22). Patient 22 showed no evidence of *TP53* mutation at diagnosis, based on >1900 next-generation sequencing reads (*expanded box). All *TP53* mutated tumors also displayed chromosome 17p loss. (bottom grid) grey, present; white, absent; cross-hatched, not relevant.

Table S4 (related to Figure 2). Combined *MYC/MYCN* amplification and P53 pathway defects predict a significantly shorter time to death following relapse

Variable		Number of cases	Univariate			Multivariate
			HR	95% CI	p value	p value
P53 pathway defect & *MYC/MYCN* amplification at relapse	Yes<br>No	7/20 (35%)<br>13/20 (65%)	8.185	1.93–34.19	**0.004**	**0.004**
MYC/MYCN amplification at relapse	Yes<br>No	7/20 (35%)<br>13/20 (65%)	8.185	1.93–34.19	**0.004**	ns
P53 pathway defect at relapse	Yes<br>No	8/20 (40%)<br>12/20 (60%)	3.692	1.26–10.78	**0.017**	ns
MYCN amplification at relapse	Yes<br>No	4/20 (20%)<br>16/20 (80%)	5.633	1.35–23.44	**0.017**	ns
Pathology at diagnosis	LCA<br>Non-LCA	3/17 (18%)<br>14/17 (82%)	6.304	1.22–32.45	**0.028**	ns
P53 pathway defect at diagnosis	Yes<br>No	4/17 (24%)<br>13/17 (76%)	2.919	0.86–9.91	0.086	ns
Ch17 defects at relapse	Yes<br>No	8/14 (57%)<br>6/14 (43%)	3.149	0.78–12.71	0.107	ns
MYC amplification at relapse	Yes<br>No	3/20 (15%)<br>17/20 (85%)	2.905	0.74–11.39	0.126	ns
Subgroup	MB_{SHH}<br>MB_{Group4}	5/13 (38%)<br>8/13 (62%)	2.272	0.67–7.67	0.186	ns
Ch17 defects at diagnosis	Yes<br>No	7/14 (50%)<br>7/14 (50%)	2.207	0.68–7.15	0.187	ns
Pathology at relapse	LCA<br>Non-LCA	5/17 (29%)<br>12/17 (71%)	1.613	0.53–4.88	0.397	ns
Metastatic stage at diagnosis	M+<br>M–	8/20 (40%)<br>12/20 (60%)	1.45	0.57–3.68	0.439	ns
Gender	Male<br>Female	12/20 (60%)<br>8/20 (40%)	0.728	0.29–1.86	0.506	ns
Ploidy at relapse	Yes<br>No	8/17 (47%)<br>9/17 (53%)	0.835	0.31–2.25	0.722	ns
Metastatic stage at relapse	M+<br>M–	10/18 (56%)<br>8/18 (44%)	0.857	0.33–2.26	0.754	ns
Ploidy at diagnosis	Yes<br>No	6/15 (40%)<br>9/15 (60%)	1.03	0.36–2.99	0.956	ns
Resection at diagnosis	STR<br>GTR	5/20 (25%)<br>15/20 (75%)	0.994	0.32–3.07	0.992	ns

Univariate and multivariate Cox proportional hazard models are shown assessing the relationship between time from relapse to death and clinical, pathological and molecular disease features, in patients receiving conventional upfront therapy. HR, hazard ratio; CI, confidence interval; LCA, large-cell/anaplastic; Ch17, chromosome 17; M+, M2+; M-, M0/M1; STR, subtotal resection; GTR, gross total resection; ns, not significant. *MYC/MYCN* amplification at diagnosis and microsatellite instability at relapse were not assessed (only one case observed).

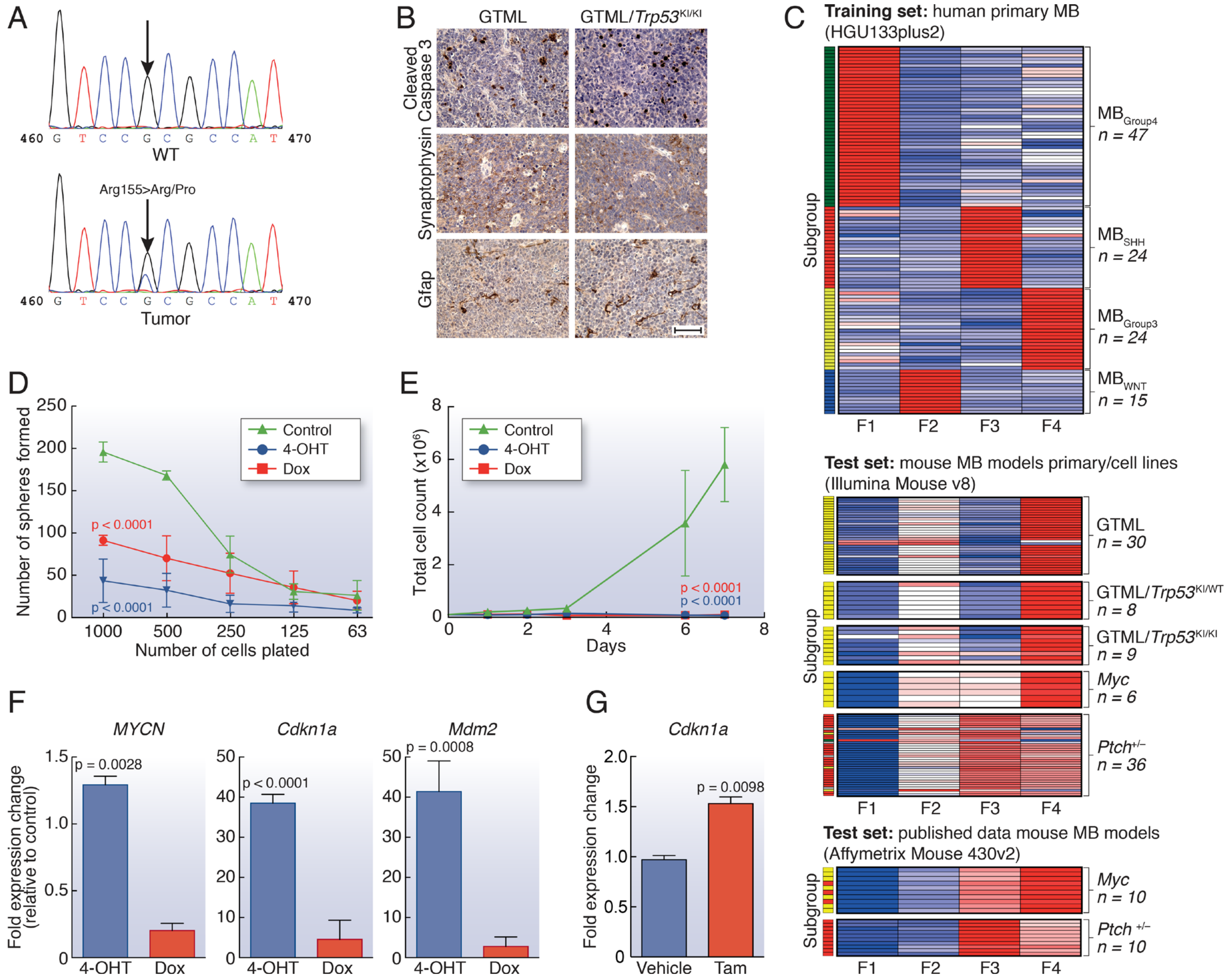

Figure S3 (related to Figure 3). Characterization of GTML/*Trp53*$^{KI/KI}$ and GTML mice

(A) Representative sequence indicates mutation in exon 5 (bottom panel), codon Arg155 leading to an amino acid change from arginine (CGC) to proline (CCC) in the tumor and not in the remaining brain tissue (top panel).

(B) H&E and immunohistochemical staining indicating apoptosis (Cleaved caspase 3), and levels of GFAP (a glial marker) and synaptophysin (a marker for neuroendocrine tumors) in GTML/*Trp53*$^{KI/KI}$ and GTML transgenic mice. Scale bar indicates 50 μm.

(C) Heatmap showing results of a classification experiment whereby human MB expression profiles were converted to 4 metagenes that describe each of the four MB sub-types using Non-negative Matrix Factorization (NMF). A Support Vector Machine (SVM) was used to create a classifier trained on the human metagenes and tested on the NMF metagenes projected from human onto mouse model expression profiles. Blue-red heatmap shows expression of subgroup specific NMF metagenes. Colored side bar shows sub-group classification calls (red, MB_{SHH}; blue, MB_{WNT}; yellow, MB_{Group3}; green, MB_{Group4}; grey, low confidence i.e. no call).

(D) Limiting dilution assay showing numbers of GTML/*Trp53*$^{KI/KI}$ neurospheres formed after treatment (3 days) with 4-OHT or dox compared to untreated control. p, two-way Anova.

(E) Limiting dilution assay showing cell growth after treatment (3 days) with 4-OHT or dox compared to untreated control. p, two-way Anova.

(F) Real-time quantitative PCR (Taqman) of human *MYCN* mRNA expression levels after dox treatment, and *Cdkn1a* and *Mdm2* mRNA expression levels after 4-OHT treatment. p, unpaired t test.

(G) Quantitative analysis of *Cdkn1a* mRNA expression in situ in tumors from GTML/*Trp53*$^{KI/KI}$ mice treated with vehicle or tamoxifen (Tam) as indicated (see Figure 3G). Expression was quantified using RNAscope SpotStudio and data are presented as relative change in the average number of spots per μm^2 of tumor cells analysed. p, unpaired t test.

Error bars represent mean ± SD.

Table S5 (related to Figure 3). *Trp53* mutations in GTML mice

		Sequence variant		Somatic *TP53* mutation count in human cancer[1]
**Mouse ID#**	**Tissue**	**cDNA**	**Protein**	
**10519**	**Tumor**	**734G>GA**	**Arg245Arg/His**	**1544 (Arg248)[2]**
10519	Brain	None detected	Wild type	n/a
**10087**	**Tumor**	**404C>CT**	**Ala135Ala/Val**	**110 (Ala138)**
10087	Brain	None detected	Wild type	n/a
**26826**	**Tumor**	**464G>GC**	**Arg155Arg/Pro**	**264 (Arg158)**
26826	Brain	None detected	Wild type	n/a
**10933**	**Tumor**	**464G>GC**	**Arg155Arg/Pro**	**264 (Arg158)**
10933	Brain	None detected	Wild type	n/a
**14658**	**Tumor**	**701T>C**	**Met234Thr**	**214 (Met237)**
14658	Brain	None detected	Wild type	n/a
**11185**	**Tumor**	**839G>T, 841A>C**	**Arg280Leu, Thr281Pro**	**103 (Arg283), 30 (Thr284)**
**14634**	**Tumor**	**382_405 het_delAATAAGCTATTCTGCCAGCTGGCG**	Wild type	n/a
**10545**	**Tumor**	**577C>CG**	**Arg193Arg/Gly**	**230 (Arg196)**
**19780**	**Tumor**	**734G>A**	**Arg245His**	**1544 (Arg248)**
**2737**	**Tumor**	**526C>T**	**His176Tyr**	**341 (His179)**
**9303**	**Tumor**	None detected	Wild type	n/a
9303	Tail	None detected	Wild type	n/a
**11153**	**Tumor**	None detected	Wild type	n/a

[1](Soussi et al. 2006). [2]Equivalent human p53 amino acids are shown in parenthesis. Ala, Alanine; Arg, Arginine; Cys, Cysteine; Gly, Glycine; His, Histidine; Leu, Leucine; Met, Methionine; Pro, Proline; Thr, Threonine; Tyr, Tyrosine; Val, Valine. n/a, not applicable.

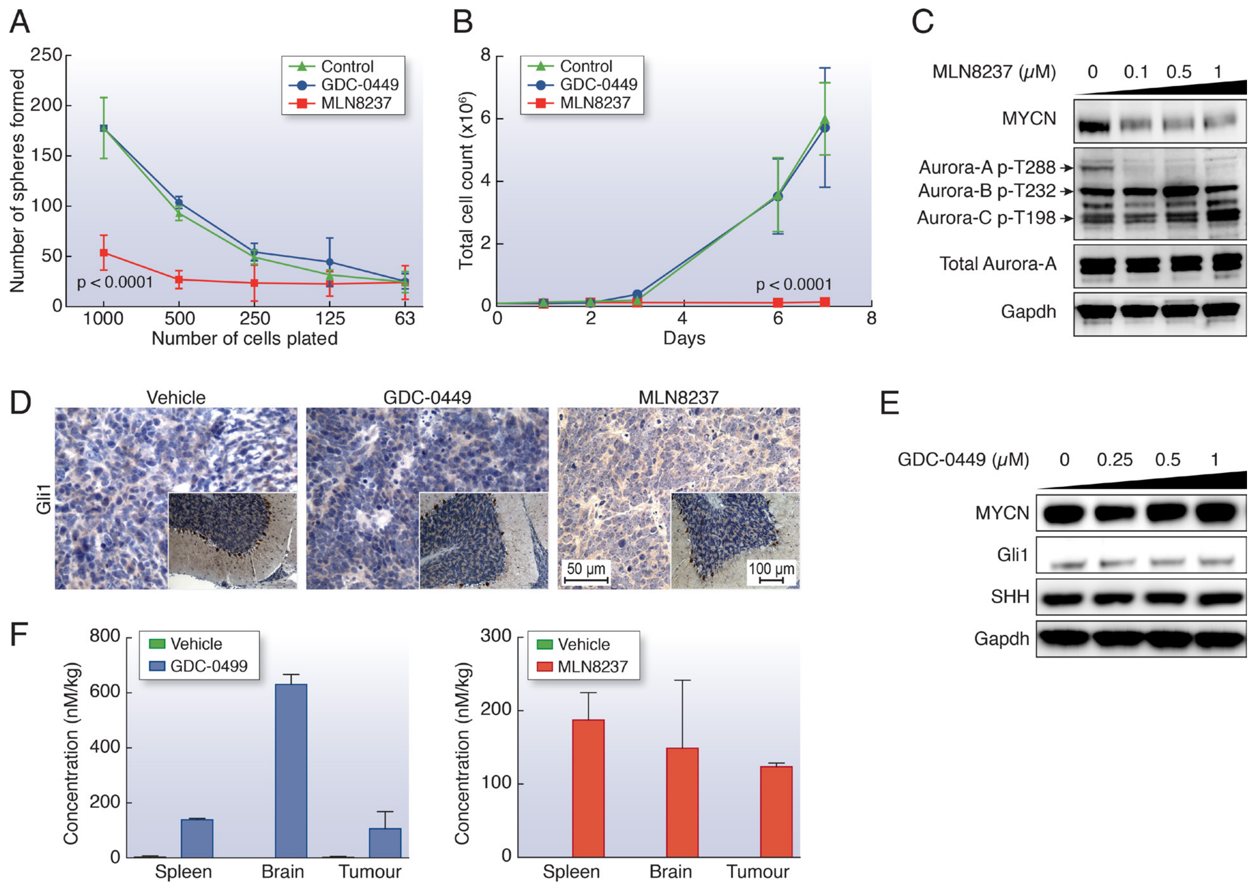

Figure S4 (related to Figure 4). Treatment with the Aurora-A kinase inhibitor MLN8237 inhibits medulloblastoma cell growth *in vitro* increases survival in GTML/*Trp53*$^{KI/KI}$ mice

(A) Limiting dilution assay showing numbers of GTML/*Trp53*$^{KI/KI}$ neurospheres formed after treatment (3 days) with GDC-0449 (blue) or MLN8237 (red) compared to untreated control (p, two-way Anova).

(B) Limiting dilution assay showing cell growth after treatment with GDC-0449 (blue) or MLN8237 (red) compared to untreated control (p, two-way Anova).

(C) Immunoblot analysis of MYCN, and phosphorylated Aurora-A (T288), Aurora-B (T232), Aurora-C (T198) and total Aurora-A protein levels in GTML/*Trp53*$^{KI/KI}$ neurospheres treated with MLN8237 at the indicated concentrations.

(D) Immunohistochemical analysis of Gli1 expression in MLN8237, GDC-0449 and vehicle treated tumor tissues. Scale bars represent 50 µm or 100 µm (inset) as indicated.

(E) Immunoblot analysis of MYCN, Gli1 and Sonic Hedgehog (SHH) protein levels in GTML/*Trp53*$^{KI/KI}$ neurospheres treated with GDC-0449 at the indicated concentrations.

(F) In vivo compound measurement of GDC-0449 and MLN8237 in spleen, brain and tumor tissue.

Error bars represent mean ± SD.

SUPPLEMENTAL EXPERIMENTAL PROCEDURES

Analysis of Molecular Subgroup Status by DNA Methylation Profiling

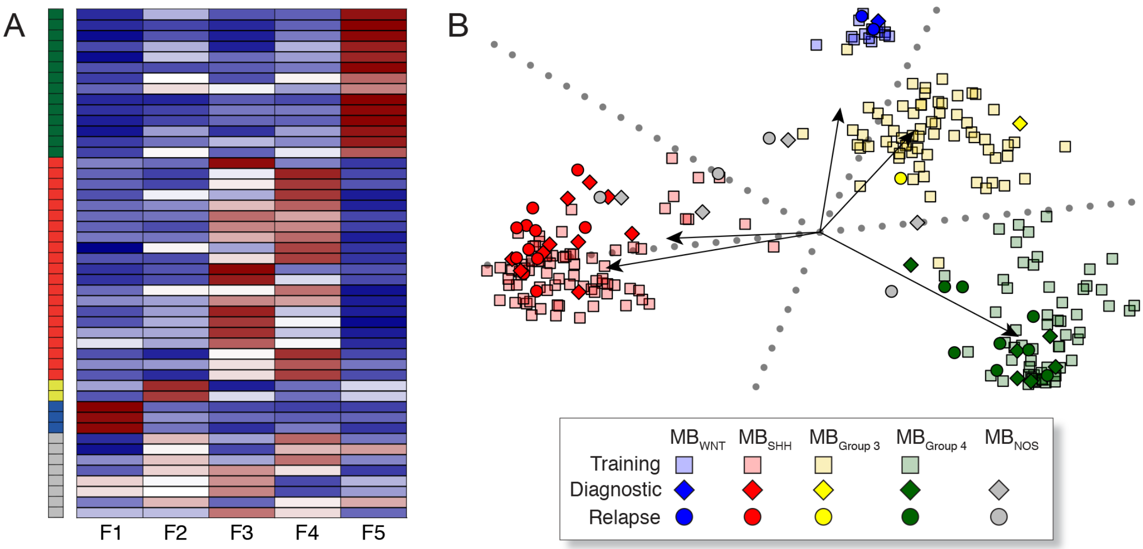

(A) Identification of subgroup membership using metagene patterns of the diagnostic and relapse samples within our study cohort. 5 metagenes (F1 to F5) were identified in the training cohort of 225 medulloblastomas using consensus NMF clustering; 2 metagenes (F3 and F4) identified the SHH subgroup, consistent with recent reports of heterogeneity within the SHH subgroup (Kool et al., 2014). Metagene projection (Tamayo et al., 2007) was employed to derive metagene values for the study cohort that were subsequently used to assign subgroup and assess classification confidence. Samples are shown in rows; samples confidently assigned to a subgroup are labelled by their assignation (red, MB_{SHH}; blue, MB_{WNT}; yellow, MB_{Group3}; green, MB_{Group4}). Samples within our study cohort that were unable to be confidently assigned (confidence score < 0.7) are labelled grey (MB_{NOS}). High, intermediate and low metagene values are shown red, white and blue, respectively.

(B) Comparison of metagene patterns for diagnostic and relapse samples within our study cohort, with metagene patterns from the training cohort. Bi-plot of principal component analysis of combined training, diagnostic and relapsing cohorts. Arrows show projections of five metagenes along first and second principal components. Individual cases are plotted by their principal component scores. Training cohort cases are shown as squares, diagnostic cohort cases as diamonds and relapsing cohort cases as circles. Additionally, cases are labelled by their assigned subgroup (red, MB_{SHH}; blue, MB_{WNT}; yellow, MB_{Group3}; green, MB_{Group4}. Cases with an unclear subgroup (confidence score < 0.7) are labelled grey (MB_{NOS})) and were not included in our subgroup analysis. Finally, training cohort cases are shown with transparency, to emphasise the members of our study cohort.

Analysis of *TP53* Status in Clinical Samples

TP53 status was assessed by direct polymerase chain reaction (PCR) based DNA sequence analysis of exons 4-9 using the following FAST PCR conditions: 95ºC for 40s; 35 cycles of 94ºC for 0s, then 64ºC for 15s; 72ºC for 45s (see below for primer sequences). Cases positive for P53 nuclear staining on immunohistochemistry (IHC) with no apparent mutation had an extended screen of exons 2-11 (see below for primer sequences). One tumor pair (patient 22, Figure S2B) that showed acquisition of a *TP53* mutation in exon 5 was assessed by next-generation sequencing using primers for exon 5 as previously described (Grossmann et al., 2011). Primer validation was performed according to manufacturer's instructions (Fluidigm) and PCR products checked using a 2100 BioAnalyzer (Agilent Technologies). Barcoded PCR products were pooled and run in a single sequencing experiment on a Roche 454 FLX sequencer (454 Life Sciences).

Primers for *TP53* PCR reaction

Location	Forward primer 5'–3'	Reverse primer 5'–3'
Exon 2	CCAGGGTTGGAAGCGTCTC	GACAAGAGCAGAAAGTCAGTCC
Exon 3/4	CATGGGACTGACTTTCTGCTC	CTTCATCTGGACCTGGGTCT
Exon 4 (part)	GGACGATATTGAACAATGGTT	ATGGAAGCCAGCCCCTCAG
Exon 4	GGCTGAGGACCTGGTCCTCTGA	GCCAGGCATTGAAGTCTCATGG
Exon 5	ATCTGTTCACTTGTGCCCTG	CAACCAGCCCTGTCGTCTCTC
Exon 6	GCCTCTGATTCCTCACTGAT	GGAGGGCCACTGACAACCA
Exon 7	AAGGCGCACTGGCCTCATCTT	CAGGGGTCAGAGGCAAGCAGA
Exon 8	GAGCCTGGTTTTTTAAATGG	TTTGGCTGGGGAGAGGAGCT
Exon 9	AGCGAGGTAAGCAAGCAGG	GCCCCAATTGCAGGTAAAACAG
Exon 10	CTTCTCCCCCTCCTCTGTTGC	GAAGGCAGGATGAGAATGGA
Exon 11	GGCACAGACCCTCTCACTCAT	TGCTTCTGACGCACACCTATT

Microsatellite Instability and Polyploidy in Clinical Samples

Microsatellite instability was assessed by a panel of markers on different chromosomes; d9s942, d9s1748 d5s346, d2s123, *MYCL*, d18s69, s10s197, *TP53*, d17s2196, d17s936, d17s969, d17s974, d17s786 and d17s1866. Tumors were classed as showing microsatellite instability if > 40% of markers analyzed were unstable compared to the diagnostic sample (Boland et al., 1998; Jung et al., 2004; Langdon et al., 2006). Polyploidy, was defined as a modal score > 2 at ≥ 2 centromeric loci examined by iFISH (chromosomes 2, 8 and 17).

Trp53 Mutational Analysis

Genomic DNA was extracted from cell lines, tumors and where available, normal brain tissue using QIAamp DNA Mini kit (Qiagen). PCR amplification of exons 5-9 was performed using primers detailed below. Products were sequenced with the original PCR primers using the BigDye Terminator Cycle Sequencing Kit and an ABI 3730 Genetic Analyzer (Applied Biosystems). Sequences were analyzed using Mutation Surveyor software (SoftGenetics).

Primers for *Trp53* PCR reaction

Location	Forward primer 5'–3'	Reverse primer 5'–3'
Exon 5/6	GATCGTTACTCGGCTTGTCC	AAGACGCACAAACCAAAACA
Exon 7	CTATAGCCAGCCATTCCCG	AGGCAGAAGCTGGGGAAG
Exon 8/9	TACACACAGTCAGGATGGGG	ATGCGAGAGACAGAGGCAAT

Real-time PCR

Total RNA was isolated from cells or tumor tissue using the miRNAeasy minikit (Qiagen) and cDNA prepared using Superscript II Reverse Transcriptase (Life Technologies). Quantitative PCR (QT-PCR) was performed in triplicate using Taqman Gene Expression mix (Life Technologies) and gene-specific primers for *Cdkn1a* (Mm04205640), *Mdm2* (Mm01233136), *MYCN* (Hs00232074) and *Actb* (Mm00607939) (Life Technologies). Relative expression was calculated according to the ΔΔCt relative quantification method against the average expression of control cells treated with ethanol or vehicle treated tumors.

In Situ Proximity Ligation Assay

Duolink in situ proximity ligation assay (PLA; Olink Bioscience) was performed on GTML/*Trp53*$^{KI/KI}$ neurospheres. Cells were fixed in 4% paraformaldehyde for 20 min, permeabilized with 0.5% Triton X-100, and blocked with 1% BSA for 30 min at room temperature followed by incubation with paired primary antibodies, MYCN (OP-13, Merck-Millipore) with Aurora-A (Genetex), overnight at 4ºC. PLA detection was performed as recommended by the manufacturer. Images were taken and analyzed using the Zeiss LSM700 confocal microscope and analyzed using DuoLink image analysis software.

Quantitative In Situ RNA Analysis

Dual color RNA *in situ* hybridization was performed using the RNAscope 2-plex Chromogenic Reagent Kit (Advanced Cell Diagnostics, ACD) according to the manufacturer's instructions. Paired double-Z oligonucleotide probes were designed against *Cdkn1a* using custom software as previously described (Wang et al., 2012). *Cdkn1a*-specific RNA target Z probe pairs (20) targeted bps 19 through 1240 of the *Cdkn1a* cDNA sequence (NM_007669.4). Probe sets specific for mouse *Ubc* (ubiquitin C), *Polr2a* (DNA-directed RNA polymerase II subunit RPB1) and *Ppib* (Peptidylprolyl Isomerase B, Cyclophilin B) and *dapB* (dihydrodipicolinate reductase) gene from *B. subtilis* were also used. FFPE tissue blocks were sectioned at 4 µm. Slides were baked for 1 hr at 60°C prior to use. After de-paraffinization and dehydration, the tissues were air dried and treated with peroxidase blocker before boiling at 100-104 °C in a pre-treatment solution for 15 min. Protease was then applied for 30 min at 40 °C. Target probes for each two-gene combination were premixed and hybridized together for 2 hr at 40 °C, followed by a series of signal amplification and washing steps. All hybridizations at 40 °C were performed in a HybEZ Hybridization System (ACD). Following the RNAscope assay, samples were counterstained for 2 minutes with 50% Gill's Hematoxylin diluted in dH_20. Hybridization signals were detected by sequential chromogenic reactions using red and green chromogens, and RNA staining signal was identified as red and green punctate dots. Each sample was quality controlled for RNA integrity with a probe specific to the *Ppib* housekeeping gene only samples with an average of >4 dots per cell were included for analysis. Negative control background staining was evaluated using a probe specific to the bacterial *dapB* gene; only samples with an average of <1 dot per 10 cells were included for analysis. *Cdkn1a* expression was quantitated using RNAscope SpotStudio (Wang et al., 2013). Outline regions of interest (ROI) were classified into individual dots and clustered dots within the ROI. Hematoxylin-stained nuclei and cell boundaries were also detected. RNAscope *Cdkn1a* mRNA images were batch analyzed after selecting appropriate ROIs manually. For each whole slide image, three ROIs were selected to represent the entire tumor section. The number of cells analyzed ranged from 4,000 to 43,000.

Pharmacokinetic analysis of MLN8237 and GDC-0449

Calibration and quality control solutions were prepared in dimethyl sulfoxide (DMSO). MLN8237 and GDC-0449 calibration standards used for spiking were prepared to give final matrix concentrations of 2-10000 nM. Quality Control (QC) standards were also prepared to give a final concentration of 25, 250, 750 and 2500 nM. A stock solution of Olomoucine (Sigma-Aldrich) internal standard (IS) was prepared in DMSO at a concentration of 1 mM and further diluted in methanol to give a working IS solution of 250 nM for quenching. Plasma and tissue samples were homogenized in either 3 or 5 ml/g phosphate buffered. 100 µl aliquots of untreated mouse plasma or tissue homogenates were spiked with 10 µl of the appropriate calibration or QC standard solutions. 100 µl aliquots of the unknown samples were spiked with 10 µl DMSO. Where necessary, plasma and tissue samples were diluted with untreated (blank) matrix. Spiked protein calibration standards, QCs and unknown samples were precipitated with 300 µL methanol containing 250 nM IS. Blank samples were prepared by spiking 100 µl untreated plasma/tissue with 10 µl DMSO and protein precipitated with 300 µl methanol. After centrifugation, supernatants were analyzed by liquid chromatography with tandem mass spectrometry detection (LC-MS/MS) using a Xevo TQ-S mass spectrometer coupled with an Acquity ultra-performance liquid chromatography UPLC H-class system (Waters). Chromatography was carried out using a Phenomenex C18 X-B column (2.6 µm, 50 mm x 2.1 mm ID) with a gradient mobile phase consisting of 0.1 % formic acid and methanol. 2ml of sample was injected on to the column using a flow rate of 0.6 ml/min with a 5 minute run time. Both analytes and IS were ionized using electrospray interface in positive ion mode. Detection was via tandem mass spectrometry (MS/MS) in multiple reaction monitoring (MRM) mode. The transitions m/z 519.12-139.04, 421.11-110.87 and m/z 299.19-177.29 were monitored for MLN8237, GDC-0449 and IS respectively. Data acquisition was performed using Targetlynx. The assay was linear over the range 2-10,000 nM.

Expression Microarray Analysis

Affymetrix HGU133plus2 expression profiles of primary tumors from 110 individuals with a diagnosis of medulloblastoma were taken from previously published studies (Fattet et al., 2009; Kool et al., 2008) (GSE12992 and GSE10327, respectively). Raw data were normalized and processed using gcRMA (Bioconductor/R). Processed data were converted to four metagenes representing the four sub-groups using non-negative matrix factorization (NMF) and these metagenes projected onto the mouse tumor/cell line expression profiles using an adaption of a previously described procedure (Tamayo et al., 2007) (scripts available upon request). Mouse expression profiles were generated using Illumina Mouse v8 arrays according to manufacturer's instructions. Raw data were processed using the beadarray package (Bioconductor/R). Expression profiles from primary murine medulloblastoma samples and cell lines included a total of 47 from GTML-based mouse models, 6 from a *Myc/Trp53*-deficient mouse model (Kawauchi et al., 2012) (gifted by Martine Roussel, St. Jude Children's Research Hospital) and 36 from *Ptch*$^{+/-}$ mice (Lastowska et al., 2013) (GSE43994). Also included were published *Ptch*$^{+/-}$ and *Myc/Trp53*-deficient mouse gene expression profiles (Kawauchi et al., 2012) (GSE34126 and GSE24628). Subgroup calls for mouse tumors were made using a support vector machine algorithm (SVM) trained on the four human subgroup metagenes and tested on the projected mouse metagenes.

SUPPLEMENTAL REFERENCES

Boland, C. R., Thibodeau, S. N., Hamilton, S. R., Sidransky, D., Eshleman, J. R., Burt, R. W., Meltzer, S. J., Rodriguez-Bigas, M. A., Fodde, R., Ranzani, G. N., and Srivastava, S. (1998). A National Cancer Institute Workshop on Microsatellite Instability for cancer detection and familial predisposition: development of international criteria for the determination of microsatellite instability in colorectal cancer. Cancer research *58*, 5248-5257.

Fattet, S., Haberler, C., Legoix, P., Varlet, P., Lellouch-Tubiana, A., Lair, S., Manie, E., Raquin, M. A., Bours, D., Carpentier, S., *et al.* (2009). Beta-catenin status in paediatric medulloblastomas: correlation of immunohistochemical expression with mutational status, genetic profiles, and clinical characteristics. The Journal of pathology *218*, 86-94.

Grossmann, V., Kohlmann, A., Zenger, M., Schindela, S., Eder, C., Weissmann, S., Schnittger, S., Kern, W., Muller, M. C., Hochhaus, A., *et al.* (2011). A deep-sequencing study of chronic myeloid leukemia patients in blast crisis (BC-CML) detects mutations in 76.9% of cases. Leukemia *25*, 557-560.

Jung, H. L., Wang, K. C., Kim, S. K., Sung, K. W., Koo, H. H., Shin, H. Y., Ahn, H. S., Shin, H. J., and Cho, B. K. (2004). Loss of heterozygosity analysis of chromosome 17p13.1-13.3 and its correlation with clinical outcome in medulloblastomas. J Neurooncol *67*, 41-46.

Kool, M., Jones, D. T., Jager, N., Northcott, P. A., Pugh, T. J., Hovestadt, V., Piro, R. M., Esparza, L. A., Markant, S. L., Remke, M., *et al.* (2014). Genome Sequencing of SHH Medulloblastoma Predicts Genotype-Related Response to Smoothened Inhibition. Cancer cell *25*, 393-405.

Kool, M., Koster, J., Bunt, J., Hasselt, N. E., Lakeman, A., van Sluis, P., Troost, D., Meeteren, N. S., Caron, H. N., Cloos, J., *et al.* (2008). Integrated genomics identifies five medulloblastoma subtypes with distinct genetic profiles, pathway signatures and clinicopathological features. PloS one *3*, e3088.

Lastowska, M., Al-Afghani, H., Al-Balool, H. H., Sheth, H., Mercer, E., Coxhead, J. M., Redfern, C. P., Peters, H., Burt, A. D., Santibanez-Koref, M., *et al.* (2013). Identification of a neuronal transcription factor network involved in medulloblastoma development. Acta Neuropathol Commun *1*, 35.

Soussi, T., Asselain, B., Hamroun, D., Kato, S., Ishioka, C., Claustres, M., and Beroud, C. (2006). Meta-analysis of the p53 mutation database for mutant p53 biological activity reveals a methodologic bias in mutation detection. Clinical cancer research : an official journal of the American Association for Cancer Research *12*, 62-69.

Wang, F., Flanagan, J., Su, N., Wang, L. C., Bui, S., Nielson, A., Wu, X., Vo, H. T., Ma, X. J., and Luo, Y. (2012). RNAscope: a novel in situ RNA analysis platform for formalin-fixed, paraffin-embedded tissues. The Journal of molecular diagnostics : JMD *14*, 22-29.

Wang, Z., Portier, B. P., Gruver, A. M., Bui, S., Wang, H., Su, N., Vo, H. T., Ma, X. J., Luo, Y., Budd, G. T., and Tubbs, R. R. (2013). Automated quantitative RNA in situ hybridization for resolution of equivocal and heterogeneous ERBB2 (HER2) status in invasive breast carcinoma. The Journal of molecular diagnostics : JMD *15*, 210-219.
