# Supplementary material for: Combined MYC and P53 Defects Emerge at Medulloblastoma Relapse and Define Rapidly Progressive, Therapeutically Targetable Disease
Source: Cancer Cell. 2015 Jan 12;27(1):72–84. doi: 10.1016/j.ccell.2014.11.002 (PMC4297293; doi:10.1016/j.ccell.2014.11.002)
Supplement: Document S2. Article plus Supplemental Information [file mmc2.pdf]

Cancer Cell
Article

Combined MYC and P53 Defects Emerge at Medulloblastoma Relapse and Define Rapidly Progressive, Therapeutically Targetable Disease

Rebecca M. Hill,[1,15] Sanne Kuijper,[2,15] Janet C. Lindsey,[1,15] Kevin Petrie,[2,15] Ed C. Schwalbe,[1] Karen Barker,[2] Jessica K.R. Boult,[3] Daniel Williamson,[1] Zai Ahmad,[2] Albert Hallsworth,[2] Sarra L. Ryan,[1] Evon Poon,[2] Simon P. Robinson,[3] Ruth Ruddle,[2] Florence I. Raynaud,[2] Louise Howell,[2] Colin Kwok,[2] Abhijit Joshi,[4] Sarah Leigh Nicholson,[1] Stephen Crosier,[1] David W. Ellison,[5] Stephen B. Wharton,[6] Keith Robson,[7] Antony Michalski,[8] Darren Hargrave,[8] Thomas S. Jacques,[8,9] Barry Pizer,[10] Simon Bailey,[1] Fredrik J. Swartling,[11] William A. Weiss,[12,13,14] Louis Chesler,[2,16,*] and Steven C. Clifford[1,16,*]

[1]Northern Institute for Cancer Research, Newcastle University, Newcastle upon Tyne NE1 4LP, UK
[2]Division of Clinical Studies
[3]Division of Radiotherapy and Imaging
The Institute of Cancer Research, Sutton SM2 5NG, UK
[4]Department of Cellular Pathology, Royal Victoria Infirmary, Newcastle upon Tyne NE1 4LP, UK
[5]St. Jude Children's Research Hospital, Memphis, TN 38105, USA
[6]Sheffield Institute for Translational Neuroscience, University of Sheffield, Sheffield S10 2HQ, UK
[7]Children's Brain Tumour Research Centre, Queen's Medical Centre, University of Nottingham, Nottingham NG7 2RD, UK
[8]Great Ormond Street Hospital for Children NHS Foundation Trust, London WC1N 3JH, UK
[9]Neural Development Unit, UCL Institute of Child Health, London WC1N 1EH, UK
[10]Oncology Unit, Alder Hey Children's Hospital, Liverpool L12 2AP, UK
[11]Department of Immunology, Genetics and Pathology, Science for Life Laboratory, Rudbeck Laboratory, Uppsala University, Uppsala 751 85, Sweden
[12]Department of Pediatrics, UCSF Benioff Children's Hospital
[13]Departments of Neurology and Neurological Surgery
[14]Helen Diller Family Comprehensive Cancer Center
University of California, San Francisco, San Francisco, CA 94158, USA
[15]Co-first author
[16]Co-senior author
*Correspondence: louis.chesler@icr.ac.uk (L.C.), steve.clifford@ncl.ac.uk (S.C.C.)
http://dx.doi.org/10.1016/j.ccell.2014.11.002
This is an open access article under the CC BY license (http://creativecommons.org/licenses/by/3.0/).

SUMMARY

We undertook a comprehensive clinical and biological investigation of serial medulloblastoma biopsies obtained at diagnosis and relapse. Combined *MYC* family amplifications and P53 pathway defects commonly emerged at relapse, and all patients in this group died of rapidly progressive disease postrelapse. To study this interaction, we investigated a transgenic model of MYCN-driven medulloblastoma and found spontaneous development of *Trp53* inactivating mutations. Abrogation of p53 function in this model produced aggressive tumors that mimicked characteristics of relapsed human tumors with combined P53-MYC dysfunction. Restoration of p53 activity and genetic and therapeutic suppression of MYCN all reduced tumor growth and prolonged survival. Our findings identify P53-MYC interactions at medulloblastoma relapse as biomarkers of clinically aggressive disease that may be targeted therapeutically.

Significance

There are currently no effective therapies for children with relapsed medulloblastoma. Although clinical and biological features of the disease at diagnosis are increasingly well understood, biopsy is rarely performed at relapse, and few biological data are available to guide more effective treatments. Here, we show that medulloblastomas develop altered biology at relapse, which is predictive of disease course and cannot be detected at diagnosis. We have discovered the emergence of P53-MYC interactions at relapse, as biomarkers of clinically aggressive relapsed disease, which can be modeled and targeted therapeutically in genetically engineered mice. These data support the incorporation of biopsy at relapse into routine clinical practice, to direct palliative care and the development of improved treatment strategies.

INTRODUCTION

Relapse following conventional treatment is the single most adverse event in medulloblastoma; over 95% of relapsing patients die, accounting for ~10% of childhood cancer deaths (Pizer and Clifford, 2009). Biological investigations have to date focused on the disease at diagnosis, where disease-wide 5 year survival rates currently stand at 60%–70% (Pizer and Clifford, 2009). These studies have shown medulloblastoma is biologically heterogeneous, comprising four molecular subgroups (WNT [MB_{WNT}], SHH [MB_{SHH}], group 3 [MB_{Group3}], and group 4 [MB_{Group4}]) with distinct clinical, pathological, and molecular features (Kool et al., 2012; Taylor et al., 2012). Moreover, disease features have been identified at diagnosis that are consistently associated with clinical outcomes. For high-risk disease, these are *MYC* gene family (*MYC*, *MYCN*) amplification, *TP53* mutation, chromosome 17 defects, large-cell anaplastic pathology, metastatic disease, and subtotal surgical resection, whereas favorable-risk disease is defined by the MB_{WNT} subgroup and desmoplastic/nodular pathology in infants (Ellison et al., 2005, 2011; McManamy et al., 2007; Northcott et al., 2012a; Pfister et al., 2009; Pizer and Clifford, 2009; Rutkowski et al., 2009; Ryan et al., 2012; Taylor et al., 2012; Zhukova et al., 2013). Together, these recent advances in understanding of the disease at diagnosis are rapidly informing the design of biologically driven phase III clinical trials aimed at improved outcomes through enhanced disease-risk stratification (Pizer and Clifford, 2009).

Patient management at relapse, however, typically focuses on quality of remaining life rather than curative strategies. This absence of suitable treatment alternatives has stemmed primarily from a lack of clinical and biological data, because biopsy is rarely performed at this stage. Consequently, this has impeded the characterization of mechanisms that drive medulloblastoma relapse, and the relevance of all the established medulloblastoma disease features in the relapsed setting, has not been investigated. Moreover, this has prevented functional validation of molecular targets using animal disease models, and their assessment as biomarkers of disease course, to support the development of more effective treatments.

We therefore assembled a clinical-trials-based cohort of patient-derived medulloblastoma biopsies sampled at relapse and aimed to undertake a comprehensive analysis of their clinical and biological characteristics, in contrast with their diagnostic counterparts. Coupled with the subsequent functional validation of specific biological features which commonly emerge at relapse (combined P53-MYC defects), using genetically engineered mouse models, we further aimed to assess their potential as biomarkers of clinically aggressive relapsed disease, and as therapeutic targets, for the improved management of patients with relapsed medulloblastoma.

RESULTS

Disease Characteristics of Relapsed Medulloblastoma

We undertook a detailed assessment of the clinical, pathological, and molecular characteristics of relapsed medulloblastoma, in a cohort of 29 recurrent tumors and their paired diagnostic samples, recruited from the recent UK Children's Cancer and Leukemia Group (CCLG) Recurrent PNET (CNS 2000 01) trial (Pizer et al., 2011) and UK CCLG treatment centers. We first assessed all molecular disease features with established significance at diagnosis including chromosome 17 and P53 pathway status (*TP53* mutation and p53 nuclear accumulation, *CDKN2A* [$p14^{ARF}$] and *MDM2* status), *MYC* gene family (*MYC*, *MYCN*) amplification, polyploidy, *CTNNB1* mutation, and molecular subgroup status (Table 1; Table S1 available online) (Ellison et al., 2011, 2005; Frank et al., 2004; Jones et al., 2012; Northcott et al., 2012a, 2012b; Pfister et al., 2009; Robinson et al., 2012; Ryan et al., 2012; Taylor et al., 2012; Zhukova et al., 2013). Only the tumor molecular subgroup was unchanged at diagnosis and relapse in all cases (Figure 1A), in agreement with the only other published cohort of medulloblastomas sampled and subgrouped at relapse (Ramaswamy et al., 2013). Subgroup distribution in the cohort of relapsed tumors sampled in our study was also consistent with Ramaswamy et al., as well as an unbiased cohort of relapsing tumors from a trial-based medulloblastoma study that were sampled at diagnosis (Schwalbe et al., 2013) (Table S2).

All other features examined showed evidence of alteration at relapse, with the majority (30/44, 68%) representing acquired high-risk disease features (Figures 1B and 1C; Table 1; Table S3) (Lannering et al., 2012; McManamy et al., 2007). Distant metastases were significantly enriched at both diagnosis and recurrence in our relapsed study cohort compared to large historic cohorts of tumors taken at diagnosis ($p < 0.003$), whereas high-risk molecular features (*MYC* and *MYCN* gene amplification, *TP53* mutation) occurred at significantly greater frequencies at relapse than at diagnosis (Figures 1B and 1C; Figures S1A–S1E; Table S3) (Pfaff et al., 2010; Pfister et al., 2009; Ryan et al., 2012). Aggressive pathology (large-cell anaplastic [LCA] variant) and *TP53* mutation were always either maintained from diagnosis to relapse or acquired at relapse. Two of two assessable *TP53* mutations tumors were somatic in origin. *TP53* mutation was identified in three of six p53-immunopositive tumors sampled at diagnosis (versus 0/17 immunonegative; $p = 0.04$, Fisher's exact test) and eight of nine immunopositive tumors sampled at relapse (versus 0/18 immunonegative; $p = 4 \times 10^{-6}$, Fisher's exact test, Table 1). Relapse following upfront radiotherapy (RT) was fatal in all cases (22/22). The only long-term survivors were infants receiving RT at recurrence (four of four, median overall survival 17 years (range 8.9–19.2 years); Figures S1F–S1H; Table 1).

Combined MYC and P53 Defects Commonly Emerge at Medulloblastoma Relapse

P53 pathway defects (*TP53* mutation, *CDKN2A* deletion) and *MYC* gene family amplification were the only disease features, which were significantly associated at relapse (Figure S2A). In patients receiving standard upfront radiotherapy and chemotherapy, these defects emerged in combination and were significantly more frequent at relapse (32% [seven of 22]) compared to diagnosis (0/19; $p = 0.01$, Fisher's exact test, Figures 2A and 2B). Single *MYC* gene family ($n = 1$) or P53 pathway aberrations ($n = 1$) were rarely observed in isolation at relapse in this treatment group (Figure 2A).

Combined P53-MYC defects characterized relapsed tumors of all molecular subgroups and occurred in combinations of specific

Table 1. Detailed Clinical, Pathological, and Molecular Characteristics of 29 Paired Medulloblastomas Sampled at Diagnosis and Relapse Showing Altered and Acquired Features at Relapse

No radiotherapy at diagnosis: patients 1–7; Radiotherapy at diagnosis: patients 8–12, 13–14, 15–16, 17–25, 26–29 (each patient: D | R)

Progression-free survival (patients 1–29): ADF, ADF, ADF, ADF, DOD, DOD, DOD, DOD, DOD, DOD, DOD, DOD, DOD, DOD, DOD, DOD, DOD, DOD, DOD, DOD, DOD, DOD, DOD, DOTC, DOD, DOD, DOD, DOTC, DOD

		Summary of demographics: Diagnosis	Summary of demographics: Relapse	Altered events	Acquired events
Molecular subgroup	Consensus subgroup / CTNNB1 mutation / Methylation subgroup: SHH	SHH 10/18 (55%)	10/22 (45%)		
	WNT	WNT 1/18 (6%)	2/22 (9%)		
	G3	G3 1/18 (6%)	1/22 (5%)		
	G4	G4 6/18 (33%)	9/22 (41%)		
Patient details	Male	18/29 (62%)	18/29 (62%)		
	Female	11/29 (38%)	11/29 (38%)		
	Infants (<4 years)	6/29 (21%)	5/29 (17%)		
Pathology variant	CLA	16/26 (61%)	14/29 (48%)	5/22 (23%) (CLA, LCA, DN)	2/22 (9%)
	LCA	3/26 (12%)	6/29 (21%)		
	DN	7/26 (27%)	4/29 (14%)		
	NOS	0/26 (0%)	5/29 (17%)		
Disease location	Local	29/29 (100%)	14/26 (54%)	17/26 (65%)	10/26 (38%)
	Distant	10/29 (34%)	17/26 (65%)		
Treatment and outcome	Complete resection	20/29 (69%)	3/29 (10%)		
	Subtotal resection	9/29 (31%)	6/29 (21%)		
	Degree unknown	0/29 (0%)	16/29 (55%)		
	Biopsy	0/29 (0%)	4/29 (14%)		
	Biopsy site				
	Craniospinal irradiation	22/29 (76%)	4/29 (14%)		
	Focal radiotherapy	0/29 (0%)	4/26 (15%)		
	Chemotherapy	26/28 (93%)	20/26 (77%)		
	Progression-free survival	0/29 (0%)	4/29 (14%)		
Molecular and cytogenic defects	*MYC/MYCN* amplification	2/25 (8%)	9/29 (31%)	9/25 (36%)	7/25 (28%)
	MYC amplification	1/25 (4%)	4/29 (14%)	4/25 (16%)	3/25 (12%)
	MYCN amplification	1/25 (4%)	5/29 (17%)	5/25 (20%)	4/25 (16%)
	P53 pathway defect	4/26 (15%)	9/29 (31%)	3/26 (12%)	3/26 (12%)
	TP53 mutation	4/26 (15%)	8/29 (28%)	2/26 (8%)	2/26 (8%)
	p53 nuclear staining	6/23 (26%)	9/27 (33%)	5/23 (22%)	3/23 (13%)
	CDKN2A homozygosity	2/24 (8%)	3/29 (10%)	0/24 (0%)	0/24 (0%)
	CDKN2A deletion	0/2 (0%)	1/3 (33%)	1/2 (50%)	1/2 (50%)
	MDM2 amplification	0/25 (0%)	0/29 (0%)		
	Ch17p FISH	8/22 (36%)	10/21 (48%)	5/20 (25%)	4/20 (20%)
	Ch17q FISH	4/22 (18%)	4/22 (18%)	2/19 (11%)	1/19 (5%)
	Polyploidy FISH	6/23 (26%)	8/24 (33%)	2/21 (10%)	2/21 (10%)
	Microsatellite instability	0/26 (0%)	1/29 (3%)	1/26 (4%)	1/26 (4%)

Demographic frequencies and altered and acquired events are shown as a proportion and percentage of the data available for each variable. D, diagnosis; R, relapse. Consensus molecular subgroup: red, SHH/MB_{SHH}; blue, WNT/MB_{WNT}; yellow, G3/MB_{Group3}; green, G4/MB_{Group4}. Pathology variant: CLA, classic; LCA, large-cell/anaplastic; DN, desmoplastic/nodular; NOS, medulloblastoma not otherwise specified. Disease location: local, M0/M1; distant, M2+. Biopsy site: gray square, primary tumor biopsied; white square, metastatic site biopsied; U, biopsy site unknown; crossed square, biopsy sample not available. Current status: ADF, alive disease-free; DOD, died of disease; DOTC, died of treatment complications. Chromosome 17 status: red, loss; green, gain. Other categories: gray square, feature present; white square, feature absent; crossed square, data not available. See also Table S1 and S2.

defects that are not observed at diagnosis. Only combined *TP53* mutation/*MYCN* amplification in MB_{SHH} have previously been observed at diagnosis (~6% of MB_{SHH}) (Zhukova et al., 2013). Direct comparison with the incidence of P53-MYC defects in our own large cohort (n = 344) of uniformly characterized primary medulloblastomas, sampled and subgrouped at diagnosis, showed significant enrichment of these combined defects in relapsed MB_{SHH} subgroup tumors following treatment with standard chemotherapy and radiotherapy (60% [three of five] versus 12% [eight of 65] at diagnosis [p = 0.0250, Figure 2B]). Equivalent trends were observed for the instances of combined P53-MYC alterations detected in relapsed MB_{WNT} and MB_{Group3} tumors (one of two tumors in both groups); these defects were not observed in any tumor sampled at diagnosis (0/48 [p = 0.0400] and 0/124 [p = 0.0159], respectively, Figure 2B). Combined defects observed at relapse in MB_{Group4} following conventional therapy were apparently less frequent than in MB_{SHH} (one of nine versus three of five; p = 0.095, Fisher's exact test). Moreover, combinations of specific P53-MYC defects were uniquely observed at relapse and were not observed at diagnosis in our large control cohorts, or in previously reported studies (Pfaff et al., 2010) (e.g., *CDKN2A* deletion and *MYC* amplification in a relapsed MB_{Group3} tumor; *TP53* mutation and *MYC* amplification in a relapsed MB_{SHH} tumor; *TP53* mutation and *MYCN* amplification in a relapsed MB_{Group4} tumor; *TP53* mutation and *MYC* amplification in a relapsed MB_{WNT} tumor).

P53 pathway and *MYC* gene family defects combined at relapse, both through maintenance of defects from diagnosis (P53 pathway) and/or the emergence at relapse (P53 pathway, *MYC* gene family) of one or both events (Figure 2C). Assessments of intratumoral molecular heterogeneity by single-cell iFISH and deep sequencing supported both de novo acquisition and clonal enrichment as mechanisms of defect emergence at relapse and demonstrated the occurrence of both defects in the same cell (Figure S2B).

P53-MYC Interactions Characterize Locally Aggressive Relapsed Disease

Importantly, the co-occurrence of P53 pathway and *MYC* gene family defects at relapse defined a population of patients with clinically aggressive tumors in which time to relapse was equivalent to that of other patients, but time to death (TTD) was significantly more rapid postrecurrence (Figure 2D; Table S4). These combined P53-MYC defects were the most significant independent predictor of TTD in multivariate survival analysis, which included tumor molecular subgroup. This group of patients all died quickly within 9 months following relapse (0.57 years [0.33–0.72 years range] median time to death post-relapse, versus 1.22 years for other tumors [0.02–2.9 years]; p = 0.0165). Moreover, *MYC*-P53 and *MYCN*-P53 defects remained significantly associated with TTD when considered in isolation against patients without combined defects (p = 0.0183 and 0.0039, respectively, log rank test).

Relapsed tumors with P53-MYC defects were significantly associated with adverse LCA pathology (Ellison et al., 2011; McManamy et al., 2007) (four of five assessable tumors, 80%, p = 0.0099, Fisher's exact test), but most did not have distant metastases (five of seven, 71%), suggesting locally aggressive disease (Figure 2E). Moreover, these tumors could not be distinguished by their clinical and pathological features and require biopsy and staging at the molecular level. In summary, our findings demonstrate that the emergence of combined *MYC* gene family amplification and P53 pathway defects is a common event at relapse following standard upfront therapy, associated with an aggressive clinical course, and can occur in tumors from all molecular disease subgroups and in specific combinations of genetic events that are not observed at diagnosis. Such patients could potentially be targeted using biomarker-driven, individualized therapeutic approaches.

Trp53 and *MYCN* Interact Directly in Medulloblastoma Development

These clinical observations and previous modeling of medulloblastoma in mice suggested that aberrant activation of the *MYC* gene family synergizes with inactivation of p53 or Rb in the genesis of biologically aggressive medulloblastoma (Kawauchi et al., 2012; Pei et al., 2012; Shakhova et al., 2006). The hypothesis that MYC or MYCN specifically interacts with p53 loss of function was established in recent studies in which *Trp53*-inactivated murine cerebellar stem or progenitor cells were transformed by forced overexpression of exogenous *Myc* or *Mycn*, driving formation of aggressive tumors resembling human medulloblastoma following transplantation into the cerebellum (Kawauchi et al., 2012; Pei et al., 2012). To investigate whether the P53-MYC interaction could be directly responsible for the genesis of spontaneous tumors within a native anatomic and developmental context, we examined *Trp53* status using a transgenic MYCN-driven mouse model (GTML; *Glt1-tTA/TRE-MYCN-Luc*) (Swartling et al., 2010). Selection of this experimental system was of particular interest given that GTML is a native transgenic model of medulloblastoma driven by fully reversible expression of *MYCN*, allowing direct assessment of its role in spontaneous tumor development. Somatic *Trp53* DNA-binding domain mutations were found in 83% of tumors examined (ten of 12) (Figure S3A; Table S5). We next tested directly whether tumor growth was dependent on both p53 and MYCN by generating GTML mice deficient in functional p53, using a mouse model in which the endogenous *Trp53* gene is replaced with a knockin allele ($Trp53^{KI}$) encoding a 4-hydroxytamoxifen (4-OHT)-regulatable $p53ER^{TAM}$ fusion protein (Christophorou et al., 2005). Mice completely deficient for p53 (GTML/$Trp53^{KI/KI}$) developed tumors with dramatically increased penetrance and significantly decreased overall survival (100%, 43/43 versus 6%, three of 50 in GTML, $p < 0.0001$, Figure 3A). Medulloblastomas from GTML/$Trp53^{KI/WT}$ and GTML/$Trp53^{KI/KI}$ mice uniformly displayed aggressive clinical and pathological features (high mitotic index, LCA pathology) equivalent to that of tumors in GTML mice with spontaneous *Trp53* mutations (Figure 3B). Moreover, tumors of all three genotypes were representative of the locally aggressive disease features (i.e., nonmetastatic, LCA) of the majority of P53-MYC-associated relapsed human tumors (Figures 2E, 3B, and S3B) and displayed gene expression profiles characteristic of human MB_{Group3} (Figures 3C and S3C).

MYCN-Driven Murine Tumor Maintenance Is Dependent on p53 and MYCN Status

Both p53 loss of function and expression of *MYCN* were required for maintenance of GTML/$Trp53^{KI/KI}$ tumors. Addition

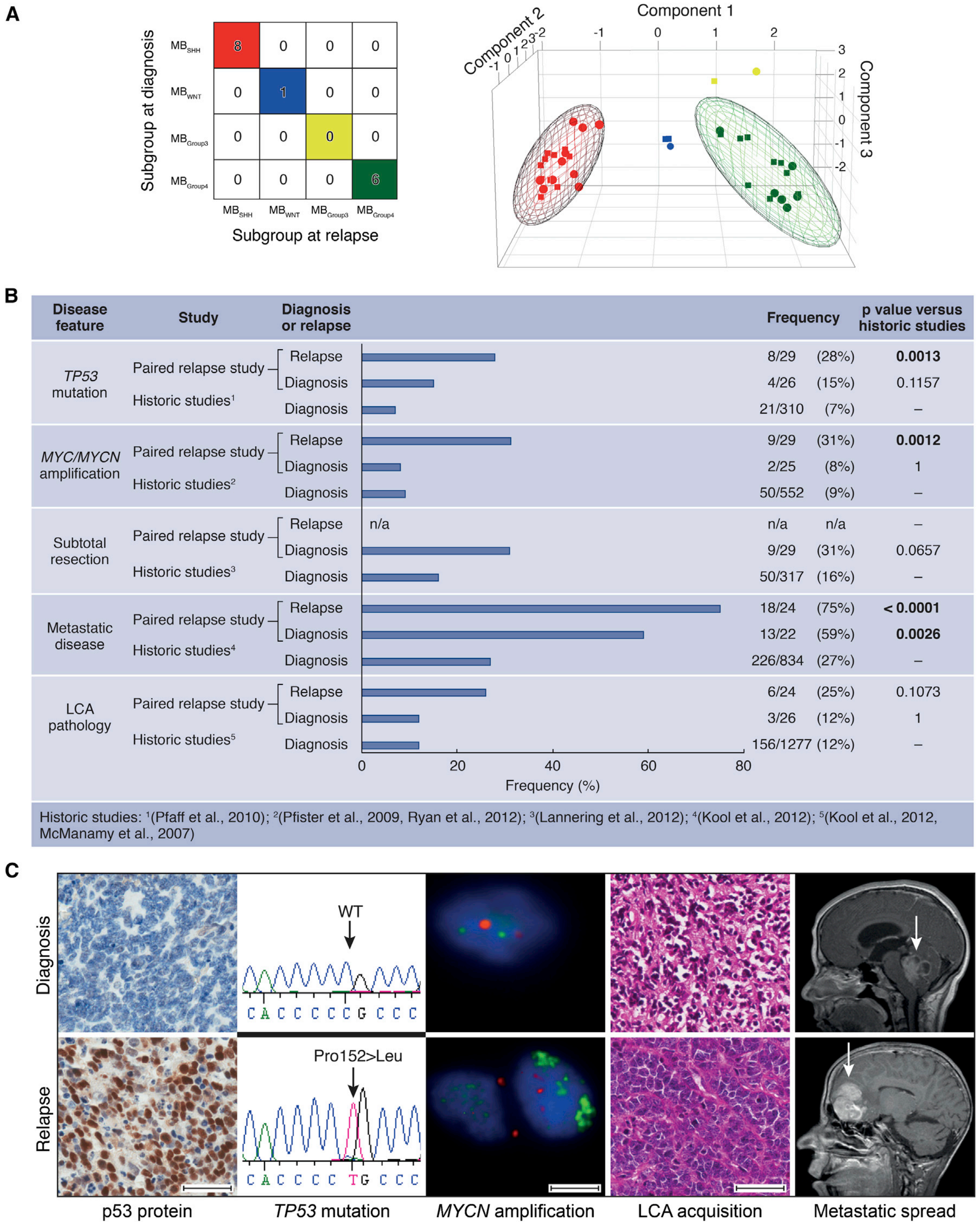

Figure 1. Relapsed Medulloblastomas Maintain the Molecular Subgroup but Are Enriched for Multiple High-Risk Clinical and Molecular Features

(A) Consensus clustering (left) and principal component analysis (PCA) (right) of medulloblastoma subgroups at diagnosis and relapse. Consensus molecular subgroups: red, MB_{SHH}; blue, MB_{WNT}; yellow, MB_{Group3}; green, MB_{Group4}. In the PCA plot, subgroups assigned at diagnosis are represented by circles, and those assigned at relapse are represented by squares.

(B) Frequency of high-risk disease features within the present paired relapse study cohort sampled at diagnosis and relapse, compared to large historic cohorts sampled at disease diagnosis. p, Fisher's exact test.

(legend continued on next page)

of either tamoxifen (Tam), which is metabolized to 4-OHT in the liver leading to reactivation of p53, or Dox (suppression of *MYCN* expression) resulted in loss of clonogenic capacity and reduced growth in GTML/*Trp53*$^{KI/KI}$ medulloblastoma-derived neurospheres, associated with loss of *MYCN* expression and induction of p53 target genes, respectively (Figures S3D–S3F). In vivo, administration of either drug led to increased survival in GTML/*Trp53*$^{KI/KI}$ mice, relating to inhibition of tumor growth (Tam) or induction of tumor regression (Dox) (Figures 3D and 3E). Treatment with either Dox or Tam led to dramatic tumor-specific reductions in the Ki-67 cellular proliferation marker, Dox-specific loss of *MYCN* expression, or Tam-specific induction of the p53 target *Cdkn1a* (Figures 3F–3H; Figure S3G). Together, these findings validate the critical dependency of MYCN-driven murine tumor growth on TP53 defects. The continued dependency on this interaction for tumor maintenance offers the potential for therapeutic intervention in relapsed human medulloblastomas.

Therapeutic Targeting with Aurora-A Kinase Inhibitors

We recently showed that small molecules that target the kinase domain of Aurora-A, a MYCN-binding protein and gatekeeper of MYCN oncoprotein stability, can induce regression and differentiation of MYCN-driven neuroblastoma (Brockmann et al., 2013), highlighting the clinical feasibility of targeting MYCN using this class of inhibitor. In vitro treatment of GTML/*Trp53*$^{KI/KI}$ medulloblastoma-derived neurospheres with the Aurora-A kinase inhibitor MLN8237 (Alisertib) destabilized MYCN via disruption of the Aurora-A/MYCN complex and caused growth inhibition comparable to doxycycline-mediated genetic suppression of *MYCN* expression (Figure 4A; Figures S4A–S4C). Consistent with their relationship to human MB_{Group3}, GTML/*Trp53*$^{KI/KI}$ tumors lack sonic hedgehog (SHH) signaling as evidenced by absence of Gli1 expression (Figure S4D). Thus, treatment with the SHH antagonist GDC-0449 (Vismodegib), which specifically targets medulloblastoma of granule cell origin driven by SHH expression, had no effect on MYCN and failed to reduce clonogenic capacity or growth of GTML/*Trp53*$^{KI/KI}$-derived medulloblastoma neurospheres (Figures S4A, S4B, and S4E). Moreover, MLN8237 but not GDC-0449 significantly prolonged survival in medulloblastoma-bearing GTML/*Trp53*$^{KI/KI}$ mice (Figure 4B). Treatment with MLN8237 completely inhibited tumor growth as measured by MRI (Figure 4C). In vivo compound measurement revealed both MLN8237 and GDC-0449 achieved blood-brain barrier penetration (Figure S4F). MLN8237 treatment led to an increase in phosphorylated histone H3 (indicative of an accumulation in G2 and mitosis due to Aurora-A inhibition) as well as specific reductions in both MYCN and Ki-67, but not an increase in cleaved caspase-3 (Figures 4D and 4E). Together, these results demonstrate the target-dependent activity of MLN8237 against GTML/*Trp53*$^{KI/KI}$ medulloblastomas and suggest clinical benefit in treating relapsed P53-MYC medulloblastoma with agents that target aberrant expression of *MYCN*.

DISCUSSION

Patients with medulloblastoma who relapse following upfront radiotherapy rarely survive, irrespective of therapy received postrecurrence (Pizer et al., 2011). Importantly, here we show that, whereas tumor subgroup did not change, clinical, pathological, and other molecular disease features were commonly altered at relapse. The emergence of combined P53-MYC gene family defects at relapse following standard upfront therapy is a common feature that occurs across disease subgroups, involves specific combinations of events not observed at diagnosis, and is associated with rapid progression to death. The validation of these combined mutations as therapeutically targetable molecular drivers of tumorigenesis in genetically engineered mice demonstrates the development of effective therapies for relapsed medulloblastoma will require strategies tailored to the unique molecular features of these tumors.

This study shows GTML/*Trp53*$^{KI/KI}$ mice to be an important model for understanding and targeting P53-MYC family interactions in medulloblastoma. Our preclinical investigations targeting Aurora-A kinase inhibition with MLN8237 in GTML/*Trp53*$^{KI/KI}$ mice, together with recent research describing CD532 (an Aurora-A inhibitor structurally distinct from MLN8237) (Gustafson et al., 2014), demonstrate proof-of-principle for indirect therapeutic targeting of MYCN in medulloblastoma and its advancement to the clinic. Establishment of their wider relevance to medulloblastoma at diagnosis, alongside other *MYC*/*MYCN* amplified and overexpressing malignancies, is paramount. Furthermore, the essential role of loss of functional p53 in GTML/*Trp53*$^{KI/KI}$ tumor growth suggests additional opportunities for intervention with emerging therapeutics that reactivate wild-type P53 by inhibiting the P53-MDM2 interaction (Carol et al., 2013; Chène, 2003; Van Maerken et al., 2014).

Our continuously collected and centrally reviewed trials-based cohort of 29 relapsed medulloblastomas is both representative of other reported relapse cohorts and reflective of the expected subgroup distribution of tumors at relapse (Table S2). Its investigation has enabled a comprehensive characterization of the clinical, pathological, and biological features of relapsed medulloblastoma and important discoveries with immediate implications for future clinical and research strategies. Although subgroup stability at relapse supports the use of diagnostic biopsy to define subgroup-directed therapies at relapse (e.g., SHH pathway inhibitors) (Rudin et al., 2009), we now understand that medulloblastomas display unique and emergent biology at relapse, which cannot be predicted at diagnosis. The identification of critical biomarkers such as P53-MYC defects in relapsed tumors will allow us, in the short term, to adapt palliative strategies tailoring therapy to predicted disease course and quality of remaining life. Looking to the future, the discovery of additional clinically relevant biomarkers will inform the further development and stratified use of targeted therapies. We particularly note

(C) Acquisition of molecular and clinical disease features between diagnosis (top) and relapse (bottom). Left to right: immunohistochemical analysis of p53 protein accumulation; *TP53* homozygous missense mutation (Pro152Leu); interphase fluorescence in situ hybridization (iFISH) showing *MYCN* amplification (green versus centromeric control (red); H&E showing LCA acquisition and magnetic resonance imaging (MRI) showing metastatic spread (arrows indicate tumor site). Scale bars, 50 μM (immunohistochemistry, H&E) or 5 μM (iFISH).
See also Figure S1 and Table S3.

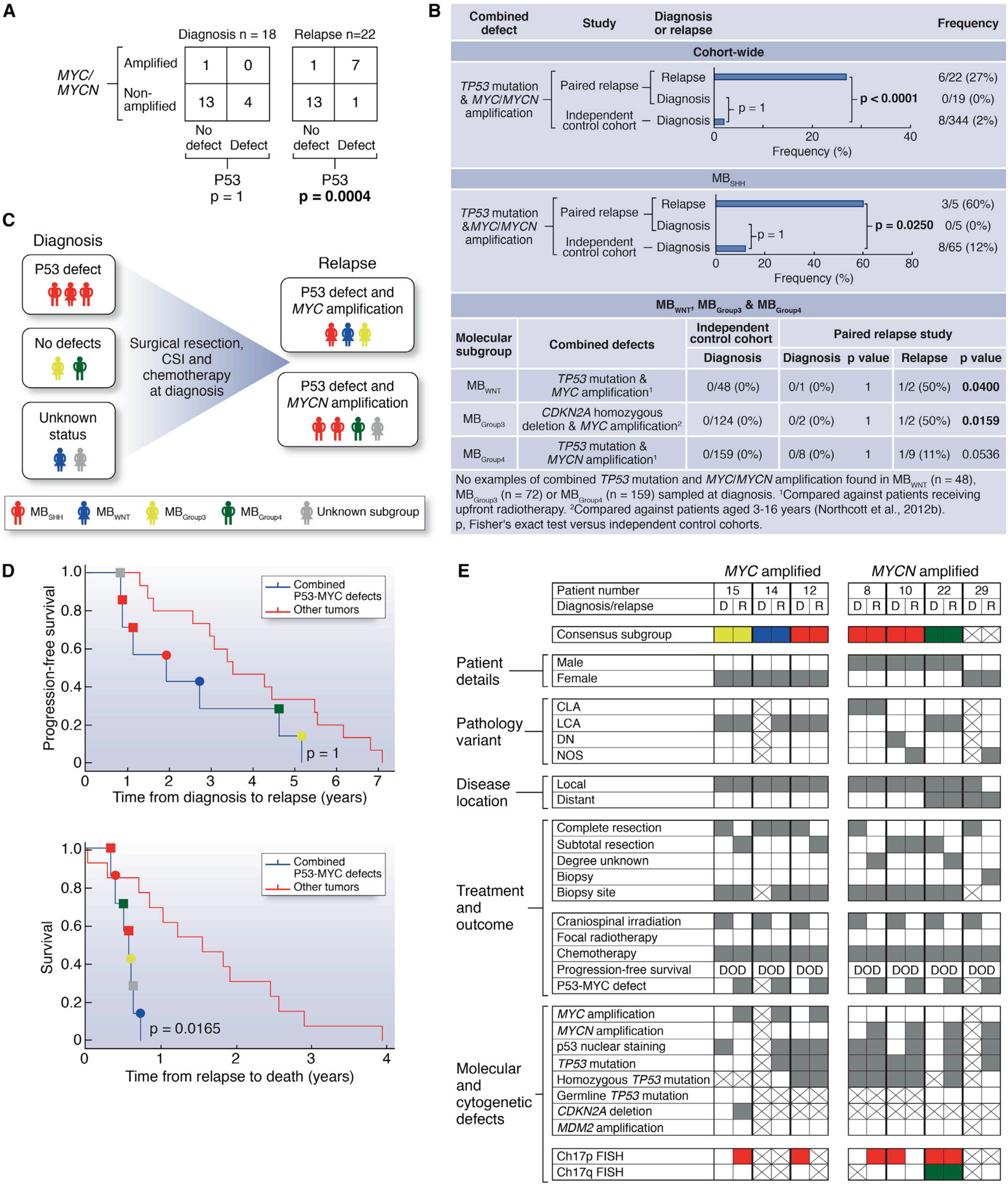

Figure 2. Combined P53 Pathway Defects and *MYC/MYCN* Amplification Commonly Emerge following Standard Upfront Radiotherapy and Chemotherapy and Correlate with Rapid Disease Progression after Relapse

(A–C) Association (A), frequency of occurrence and distribution within molecular subgroups (B), and patterns of emergence (C), of combined P53 pathway defects and *MYC/MYCN* amplification at diagnosis and relapse.

(legend continued on next page)

MB_{Group3} tumors are less commonly sampled at medulloblastoma relapse (this study; Ramaswamy et al., 2013), likely reflecting their associated early, disseminated pattern of relapse (Ramaswamy et al., 2013) and a clinical decision not to biopsy. The routine sampling of relapsed medulloblastoma is therefore now essential to expand our findings, inform comprehensive biological investigations across all clinical and molecular disease demographics, and direct clinical management and future therapeutic advances aimed at improved outcomes for children with relapsed medulloblastoma.

EXPERIMENTAL PROCEDURES

Tumor Material and Clinical Data

Clinical data and tumor tissue were obtained for 29 patients from UK CCLG institutions and collaborating centers (Table 1), encompassing patients enrolled on the Recurrent PNET (CNS 2000 01) trial (Pizer et al., 2011). The median age at diagnosis was 8.6 years (range 0.1–33.7 years), and median age of recurrence was 10.7 years (range 2.4–36.3 years) with a median time to relapse of 2.6 years (range 0.5–7.1 years). Within the cohort, six of 29 (21%) children at diagnosis were infants (<4 years old). Metastatic stage was determined according to Chang's criteria and pathology was centrally reviewed by a panel of neuropathologists from UK Children's Cancer and Leukemia Group (CCLG) according to current WHO criteria (Chang et al., 1969; Louis et al., 2007). Clinical data were collated and centrally reviewed. Genomic DNA was extracted using standard methods, and validation of paired sample identity was performed using a panel of microsatellite markers (see below). Human tumor samples were provided by the UK CCLG as part of CCLG-approved biological study BS-2007-04; informed consent was obtained from all subjects. Human tumor investigations were conducted with approval from Newcastle/North Tyneside Research Ethics Committee (study reference 07/Q0905/71).

Selection and Assessment of Critical Medulloblastoma Molecular Features

Established medulloblastoma molecular features, with validated relationships to disease molecular pathology and prognosis, were assessed. These comprised (1) the four consensus medulloblastoma molecular subgroups associated with distinct molecular events, clinicopathological features, and prognosis (Taylor et al., 2012); (2) *MYC* and *MYCN* amplification (predominant in MB_{Group3} and $MB_{SHH/Group4}$, respectively), and associated with poor outcome (Ellison et al., 2011; Northcott et al., 2012a; Pfister et al., 2009; Pizer and Clifford, 2009; Ryan et al., 2012); (3) *TP53*, one of the most frequently mutated genes in medulloblastoma, associated with $MB_{WNT/SHH}$, and reduced survival rates in the MB_{SHH} subgroup (Northcott et al., 2012a; Zhukova et al., 2013); (4) additional defects of the P53 pathway (*CDKN2A* deletion/methylation, *MDM2* amplification, and p53 nuclear accumulation) linked to poor outcome in other pediatric embryonal tumors including relapsed neuroblastoma (Carr-Wilkinson et al., 2010; Frank et al., 2004); (5) *CTNNB1* mutation, associated with MB_{WNT} (Taylor et al., 2012); (6) polyploidy, associated with genomic instability, MB_{Group3}/MB_{Group4} and poor prognosis (Jones et al., 2012; Northcott et al., 2012a); and (7) defects of chromosome 17, including the most common medulloblastoma cytogenetic abnormalities (i.e., gains of 17q, isochromosome 17q [i{17q}], and loss of 17p; Ellison et al., 2011; Pfister et al., 2009; Taylor et al., 2012), associated with MB_{Group3}/MB_{Group4} and poor survival (Pfister et al., 2009; Shih et al., 2014).

Molecular Subgroup Status

All samples, where DNA was of sufficient quantity and quality as assessed by PicoGreen dsDNA quantitation assay (Life Technologies), were processed on the 450K methylation array (Illumina). Subgrouping according to methylation status was achieved using established methods (Hovestadt et al., 2013; Schwalbe et al., 2013). Consensus nonnegative matrix factorization (NMF) clustering of a 225 member primary medulloblastoma training cohort was used to define four methylation-dependent disease subgroups by identifying subgroup-specific metagenes. A support vector machine (SVM) classifier to assign subgroup for additional diagnostic and relapsed medulloblastoma samples, based on their projected metagene profiles (Tamayo et al., 2007), was developed using previously published methods (Schwalbe et al., 2013). Confidence of the classifier call made for these samples was assessed by repeated sampling of 80% of the training cohort to rederive the classifier. Mutational analysis of *CTNNB1* (Table S1) (Taylor et al., 2012) was performed as previously described (Ellison et al., 2005, 2011) (see Supplemental Experimental Procedures).

Copy-Number Analysis in Clinical Samples

Copy-number estimates were carried out using iFISH, microsatellite typing, or multiplex ligation-dependent probe amplification (MLPA) using SALSA reagents (MRC-Holland). Copy-number assessment by iFISH of *MYC* (8q24.21 probes), *MYCN* (2p24.3 probes), and chromosome 17 imbalances (17p13.3 and 17q12 probes) versus respective centromeric reference loci was performed on available material as previously described (Lamont et al., 2004; Langdon et al., 2006; Nicholson et al., 2000). One hundred nonoverlapping nuclei were scored by two independent assessors, and amplification was defined as previously reported (Ryan et al., 2012).

Copy-number assessment by MLPA of *MYC*, *MYCN*, and *MDM2* were measured relative to four independent reference loci (*B2M*, *TBP*, 7q31, and 14q22). Normal diploid control samples were used to define cutoffs for the detection of elevated copy numbers (>95% confidence interval of the normal distribution). Tumor samples showing reproducibly elevated copy numbers (in multiple replicates and versus three or more reference loci) were deemed to have copy-number elevation. Samples with evidence of raised copy number by MLPA were validated by iFISH on available material against a panel of normal copy-number tumor controls.

Copy-number analysis of *CDKN2A* ($p14^{ARF}$) was performed using polymorphic microsatellite markers for chromosome 9p21 (d9s942 and d9s1748) as previously reported (Randerson-Moor et al., 2001). Copy-number status of three cases homozygous for both polymorphic microsatellite markers, suggestive of chromosomal deletion at the *CDKN2A* locus (Berggren et al., 2003), was further assessed by 450K methylation array (Sturm et al., 2012) (n = 2) or the Illumina Human Omniexpress array (Illumina (n = 1). Methylation of *CDKN2A* was also assessed by 450K methylation array.

Analysis of *TP53* Status in Clinical Samples

Immunohistochemistry (IHC) in human samples for p53 immunopositivity, previously associated with *TP53* mutation (Pfaff et al., 2010; Tabori et al., 2010), was performed on formalin-fixed, paraffin-embedded (FFPE) samples (M7001, Dako) using the Menapath Polymer HRP Detection system (A. Menarini Diagnostics). All samples were analyzed by a neuropathologist, blind to mutation status, and by a nuclear stain algorithm (Spectrum, Aperio Technologies). *TP53* mutation status was assessed by direct PCR-based DNA sequence analysis, and one tumor pair was assessed by next-generation sequencing (see Supplemental Experimental Procedures).

(D) Survival of patients with tumors harboring combined P53-*MYC* gene family defects versus other tumors, showing time from diagnosis to relapse (top) and relapse to death (bottom). Circle, P53-*MYC*; square, P53-*MYCN*. p, log rank test, Bonferroni corrected.
(E) Detailed clinical, pathological, and molecular demographics of patients with combined P53-*MYC* gene family defects at relapse. D, diagnosis; R, relapse. Consensus molecular subgroup (red, MB_{SHH}; blue, MB_{WNT}; yellow, MB_{Group3}; green, MB_{Group4}). Pathology variant: CLA, classic; LCA, large-cell/anaplastic; DN, nodular/desmoplastic; NOS, medulloblastoma not otherwise specified. Disease location: local, M0/M1; distant, M2+. Biopsy site: gray square, primary tumor biopsied; white square, metastatic site biopsied; crossed square, biopsy sample not available. Current status: DOD, died of disease. Chromosome 17 status: red, loss; green, gain. Other categories: gray square, feature present; white square, feature absent; crossed square, data not available.
See also Figure S2 and Table S4.

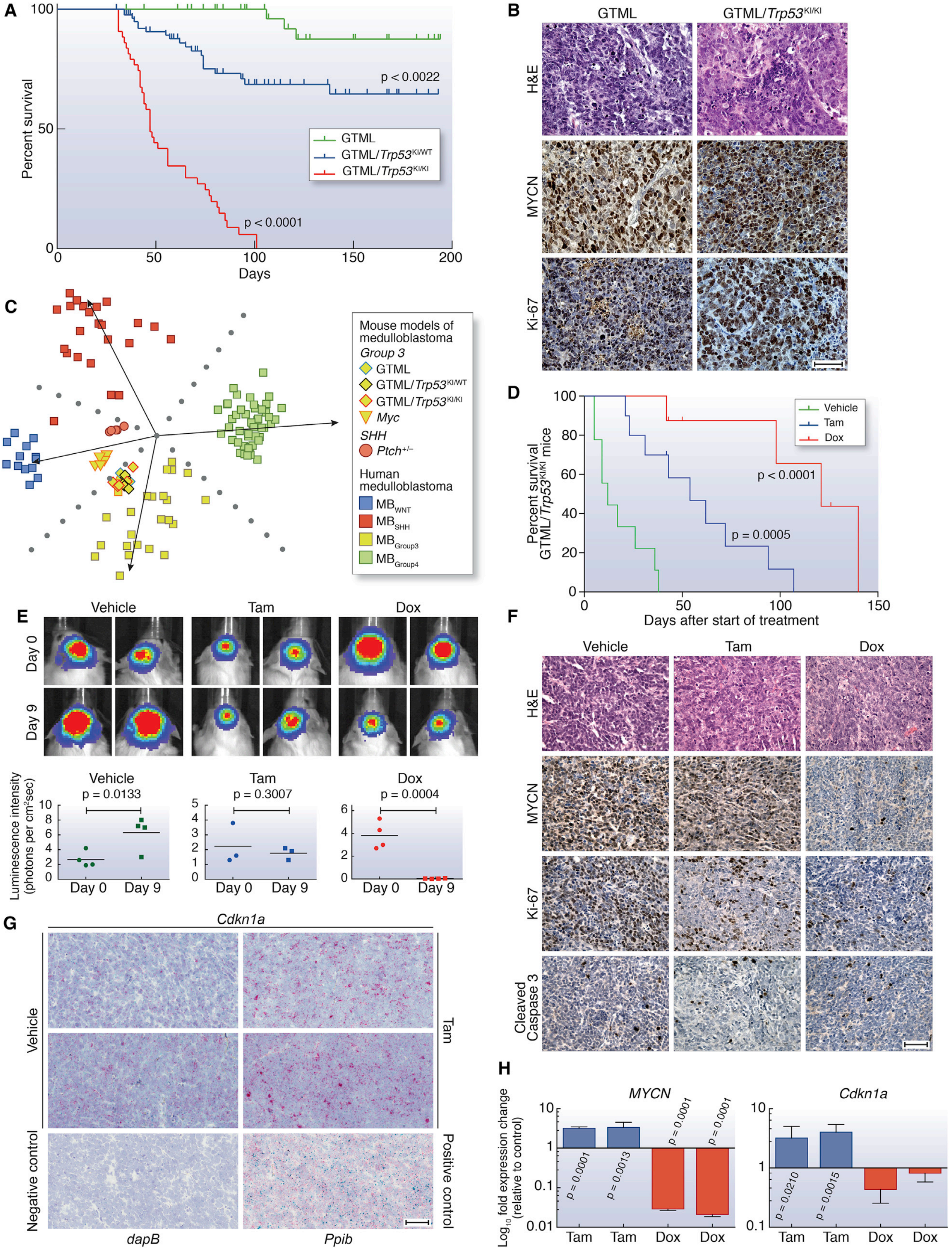

(legend on next page)

Statistical Analysis of Clinical Samples
Chi-square and Fisher's exact tests were used to assess associations between clinicopathological and molecular features, and p values were corrected for multiple testing using the Bonferroni procedure (Abdi, 2007). The log rank test was used to assess all univariate survival markers. Cox proportional hazards models were used to investigate the significance of variables for event-free survival (EFS), overall survival (OS), and time to death (TTD) analyses in (1) univariate and (2) multivariate models using forward likelihood-ratio testing.

In Vivo Studies
All experimental protocols were monitored and approved by The Institute of Cancer Research Animal Welfare and Ethical Review Body, in compliance with guidelines specified by the UK Home Office Animals (Scientific Procedures) Act 1986 and the United Kingdom National Cancer Research Institute guidelines for the welfare of animals in cancer research (Workman et al., 2010). GTML mice have been described previously (Swartling et al., 2010). The *Trp53*$^{KI/KI}$ mice were kindly provided by G.I. Evan (Christophorou et al., 2005) and crossed with GTML animals into a background of the FVB/NJ inbred strain (Taketo et al., 1991). To image for bioluminescence expression, animals were injected with 75 mg/kg D-luciferin in saline (PerkinElmer) prior to imaging in the IVIS Lumina (PerkinElmer) using Living Image Software. Transgenic GTML/*Trp53*$^{KI/KI}$ animals with bioluminescence signals higher than 1.5×10^{-9} photons/seconds (20–30 days of life) were randomized to treatment groups and treated with 30 mg/kg MLN8237 (Alisertib, Millennium) or 50 mg/kg GDC-0449 (Vismodegib, LC Laboratories). MLN8237, GDC-0449, and the respective vehicles were dosed orally on a daily basis. Doxycycline was given via chow at 1,250 mg/kg diet to provide a daily dose of approximately 160 mg/kg. Restoration of wild-type p53 was achieved by administration of either 1 mg of tamoxifen dissolved in 100 μl peanut oil carrier daily by intraperitoneal injection or via chow at 400 mg/kg diet to provide a daily dose of approximately 64 mg/kg. Animals were monitored twice a week for bioluminescence signal and were sacrificed upon detection of a signal higher than 9×10^{-9} photons/second or overt signs of intracranial expansion associated with tumor growth. Mice were allowed access to food and water ad libitum.

In Vivo Imaging
Multislice ^{1}H MRI was performed on a 7T horizontal bore microimaging system (Bruker Instruments) using a 3 cm birdcage coil and a 2.5×2.5 cm field of view. Anesthesia was induced with a 10 ml/kg intraperitoneal injection of fentanyl citrate (0.315 mg/ml) plus fluanisone (10 mg/ml, Hypnorm, Janssen Pharmaceutical), midazolam (5 mg/ml, Hypnovel, Roche), and sterile water (1:1:2). Core body temperature was maintained by warm air blown through the magnet bore. Magnetic-field homogeneity was optimized by shimming over the entire brain using an automated shimming routine (FASTmap). T_2-weighted images acquired using a rapid acquisition with refocused echoes (RARE) sequence (12 contiguous 1 mm sagittal slices or 20 contiguous 1 mm axial slices, 256×256 matrix, four averages, echo times [TE] = 36 and 132 ms, repetition time [TR] = 4.5 s, RARE factor = 8) were used for localization of the tumor and measurement of tumor volume.

Neurosphere Isolation and Culture
Tissue isolated from GTML/*Trp53*$^{KI/KI}$ tumors was transferred into cold HBSS, cut into 2–3 mm^2 pieces and dissociated before trituration in medium and filtration through 70 μm mesh. To generate neurospheres, cells were cultured under self-renewal conditions in DMEM/F12 medium (Life Technologies) supplemented with 2% B27 supplement (Life Technologies), 20 ng/ml epidermal growth factor (Sigma-Aldrich), and 20 ng/ml fibroblast growth factor (Life Technologies). For in vitro analyses, cells were treated with the following drug concentrations: 100 nM 4-OHT (Sigma-Aldrich), 1 μg/ml doxycycline (Sigma-Aldrich), 100 nM MLN8237, and 500 nM GDC-0449. Neurosphere formation was assessed by performing limiting dilutions from 1,000 to 60 cells and imaging using a Celigo S Imaging Cell Cytometer (Brooks Life Science Systems).

Western Blot Analysis and IHC
Western blot analysis of mouse tissues and neurospheres was performed as previously described (Brockmann et al., 2013; Chesler et al., 2006). For IHC of mouse tissues, samples were fixed in 4% paraformaldehyde in phosphate buffered saline for at least 24 hr, decalcified with 0.3 M EDTA, and processed using a Leica ASP300S tissue processor. Sections were cut at 4 μM for hematoxylin and eosin staining (H&E) staining and immunohistochemistry as previously described (Chesler et al., 2006). Antibodies used were MYCN (OP-13, Merck-Millipore), Ki-67 (556003, BD Biosciences), GFAP (Z0334, DAKO), Cleaved Caspase 3 (9664, Cell Signaling Technology), Synaptophysin (180130, Life Technologies), Phospo-S10-Histone H3 (9706, Cell Signaling), phospho-AurkABC (2914, Cell Signaling), AurkA (4718, Cell Signaling), Sonic Hedgehog (ab73958, Abcam), Gli-1 (2534, Cell Signaling), and GAPDH (2118, Cell Signaling).

ACCESSION NUMBERS

The Gene Expression Omnibus (GEO) accession number for 450K DNA methylation array profiles used for the determination of human medulloblastoma molecular subgroup status is GSE62618. The GEO accession number for microarray expression profiles of mouse medulloblastomas is GSE62625. The GEO SuperSeries accession number for this study is GSE62626.

SUPPLEMENTAL INFORMATION

Supplemental Information includes Supplemental Experimental Procedures, four figures, and five tables and can be found with this article online at http://dx.doi.org/10.1016/j.ccell.2014.11.002.

AUTHOR CONTRIBUTIONS

L.C. and S.C.C. conceived the study. S.L.N. and S.C. collected and processed human tissue cohorts. D.H., B.P., D.W.E., and A.M. provided human tumor

Figure 3. Aberrant Expression of MYCN in Combination with p53 Loss of Function Drives Highly Penetrant and Aggressive Medulloblastoma
(A) Kaplan-Meier survival curves for GTML/*Trp53*$^{KI/KI}$ (n = 43), GTML/*Trp53*$^{KI/WT}$ (n = 83), or GTML transgenic mice (n = 50) mice as indicated. p, log rank test.
(B) H&E and immunohistochemical staining indicating levels of MYCN protein and cell proliferation (Ki-67) in GTML/*Trp53*$^{KI/KI}$ and GTML transgenic mice.
(C) Subgroup classification of mouse expression profiles using a support vector machine trained on human medulloblastoma expression profiles and nonnegative matrix factorization for cross-species projection.
(D) Kaplan-Meier survival for GTML/*Trp53*$^{KI/KI}$ mice treated with doxycycline (Dox, n = 8) or tamoxifen (Tam, n = 10) compared to vehicle (n = 9) as indicated. p, log rank test.
(E) GTML/*Trp53*$^{KI/KI}$ mice coexpressing firefly luciferase (FLuc) were treated with Tam, Dox, or vehicle for 9 days. Bioluminescent imaging of GTML/*Trp53*$^{KI/KI}$ mice after 9 days treatment with Dox or Tam as indicated (top). Luminescence intensity at days 0 and 9 are shown (bottom). Data points represent individual mice. p, unpaired t test.
(F) H&E and immunohistochemical staining indicating levels of MYCN, Ki-67, or apoptosis (cleaved caspase 3) in GTML/*Trp53*$^{KI/KI}$ mice after treatment with Dox or Tam.
(G) RNAscope 2-plex chromogenic assay. *Cdkn1a* expression (red) was analyzed on brain sections from GTML/*Trp53*$^{KI/KI}$ mice treated with either Tam or vehicle control as indicated. Samples were costained for expression of the *Ubc* (ubiquitin C) housekeeping gene (green). Expression of *Ppib* (Peptidylprolyl Isomerase B, Cyclophilin B) (red) and *Polr2a* (DNA-directed RNA polymerase II subunit RPB1) (green) were used as positive controls. Expression of *dapB* (dihydrodipicolinate) reductase gene from *B. subtilis* was used as a negative control. Sections were counterstained with Gill's hematoxylin.
(H) Fold difference of human *MYCN* or mouse *Cdkn1a* mRNA levels in tumor tissues treated with either Dox or Tam. (p, unpaired t test.)
Scale bars, 50 μm. Error bars represent mean ± SD. See also Figure S3 and Table S5.

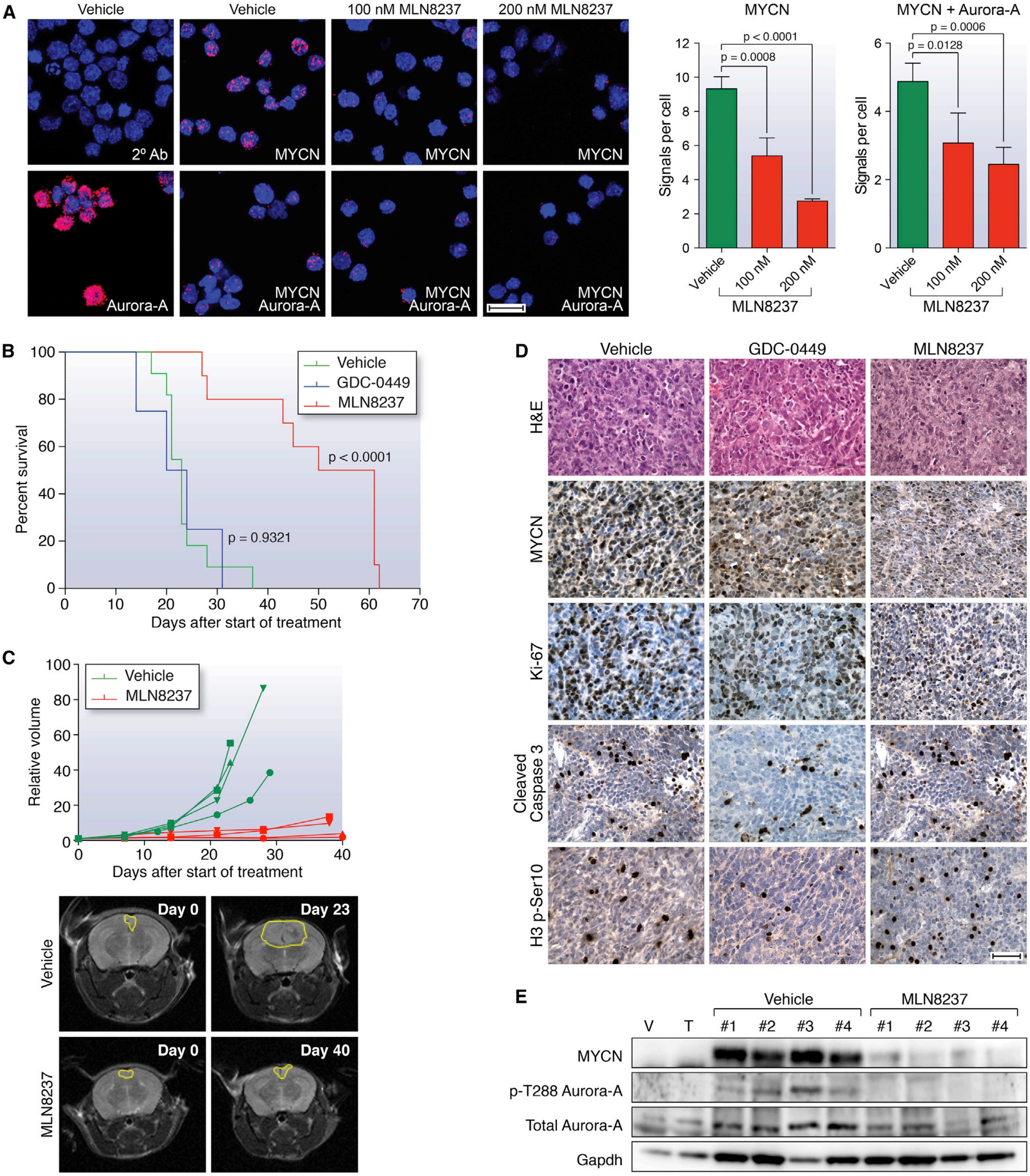

Figure 4. Therapeutic Targeting of the MYCN/Aurora-A Interaction Inhibits Tumor Growth and Prolongs Survival in GTML/*Trp53*$^{KI/KI}$ Mice

(A) Proximity ligation assay (PLA) analyzing MYCN/Aurora-A complexes in GTML/*Trp53*$^{KI/KI}$ neurospheres following MLN8237 treatment (48 hr). Left panel shows close proximity (<40 nm) of antibody conjugated PLA probes that have been ligated, amplified, and detected with complementary fluorescent probes. Red dots represent the presence of MYCN or Aurora-A protein, or MYCN/Aurora-A interactions as indicated. Antibodies used are indicated by white text (2° Ab, secondary antibody control). Scale bar, 20 μm. Right panel shows mean values of signals (red dots) per cell representing MYCN expression or MYCN/Aurora-A interactions. Values are derived from triplicate biological replicates, and error bars represent SDs. p, unpaired t test.

(legend continued on next page)

samples. S.B., S.C.C., and R.M.H. collected and centrally reviewed clinical data. R.M.H., J.C.L., and S.C.C. designed experiments on human tumor cohorts, which were carried out by R.M.H., J.C.L., and S.L.R. E.C.S. planned and executed human 450K methylation array analysis. R.M.H., J.C.L., E.C.S., and S.C.C. planned and carried out all other analyses of human tumor data. T.S.J., K.R., and S.B.W. performed central pathology review of human tumors. A.J. performed p53 immunohistochemistry analysis. S.K., K.P., F.J.S., W.A.W., and L.C. planned mouse experiments, which were carried out by S.K. and A.H. MRI of tumors was planned by J.K.R.B. and S.P.R. and carried out by J.K.R.B. In vivo compound measurement was planned by R.R. and F.I.R. and carried out by R.R. S.K., K.P., and L.C. planned experiments to characterize tumor biology and response to therapeutics, which were executed by S.K., K.B., Z.A., E.P., L.H., and C.K.; S.K., K.P., and L.C. analyzed these data. D.W. planned and executed gene expression analysis of mouse tumors. Histopathological analysis of mouse tumors was performed by T.S.J. K.P., R.M.H., L.C., and S.C.C. wrote the manuscript.

ACKNOWLEDGMENTS

This study was supported by grants from Cancer Research UK (grants C34648/A12054, C8464/A13457, and C1060/A10334), Action Medical Research (RTF1414), Sparks (09NCL02), The Brain Tumour Charity (grants SDR004X and 16/164), the JGW Patterson Foundation, and Christopher's Smile (CXC002H) and was undertaken as part of the INSTINCT network, co-funded by The Brain Tumour Charity, Great Ormond Street Children's Charity, and Children with Cancer UK (grant 16/193). T.S.J. is supported by the National Institute for Health Research and a Great Ormond Street Hospital UCL Biomedical Research Centre award. F.I.R. and R.R. are employees of the Institute of Cancer Research and are involved in the development of aurora kinase inhibitors.

Received: April 25, 2014
Revised: September 2, 2014
Accepted: November 5, 2014
Published: December 18, 2014

REFERENCES

Abdi, H. (2007). Bonferroni and Sidak corrections for multiple comparisons. In Encyclopedia of Measurement and Statistics, N.J. Salkind, ed. (Thousand Oaks, CA: Sage).

Berggren, P., Kumar, R., Sakano, S., Hemminki, L., Wada, T., Steineck, G., Adolfsson, J., Larsson, P., Norming, U., Wijkstrom, H., and Hemminki, K. (2003). Detecting homozygous deletions in the CDKN2A(p16(INK4a))/ARF(p14(ARF)) gene in urinary bladder cancer using real-time quantitative PCR. Clin. Cancer Res. *9*, 235–242.

Brockmann, M., Poon, E., Berry, T., Carstensen, A., Deubzer, H.E., Rycak, L., Jamin, Y., Thway, K., Robinson, S.P., Roels, F., et al. (2013). Small molecule inhibitors of aurora-a induce proteasomal degradation of N-myc in childhood neuroblastoma. Cancer Cell *24*, 75–89.

Carol, H., Reynolds, C.P., Kang, M.H., Keir, S.T., Maris, J.M., Gorlick, R., Kolb, E.A., Billups, C.A., Geier, B., Kurmasheva, R.T., et al. (2013). Initial testing of the MDM2 inhibitor RG7112 by the Pediatric Preclinical Testing Program. Pediatr. Blood Cancer *60*, 633–641.

Carr-Wilkinson, J., O'Toole, K., Wood, K.M., Challen, C.C., Baker, A.G., Board, J.R., Evans, L., Cole, M., Cheung, N.K., Boos, J., et al. (2010). High Frequency of p53/MDM2/p14ARF Pathway Abnormalities in Relapsed Neuroblastoma. Clin. Cancer Res. *16*, 1108–1118.

Chang, C.H., Housepian, E.M., and Herbert, C., Jr. (1969). An operative staging system and a megavoltage radiotherapeutic technic for cerebellar medulloblastomas. Radiology *93*, 1351–1359.

Chène, P. (2003). Inhibiting the p53-MDM2 interaction: an important target for cancer therapy. Nat. Rev. Cancer *3*, 102–109.

Chesler, L., Schlieve, C., Goldenberg, D.D., Kenney, A., Kim, G., McMillan, A., Matthay, K.K., Rowitch, D., and Weiss, W.A. (2006). Inhibition of phosphatidylinositol 3-kinase destabilizes Mycn protein and blocks malignant progression in neuroblastoma. Cancer Res. *66*, 8139–8146.

Christophorou, M.A., Martin-Zanca, D., Soucek, L., Lawlor, E.R., Brown-Swigart, L., Verschuren, E.W., and Evan, G.I. (2005). Temporal dissection of p53 function in vitro and in vivo. Nat. Genet. *37*, 718–726.

Ellison, D.W., Onilude, O.E., Lindsey, J.C., Lusher, M.E., Weston, C.L., Taylor, R.E., Pearson, A.D., and Clifford, S.C. (2005). beta-Catenin status predicts a favorable outcome in childhood medulloblastoma: the United Kingdom Children's Cancer Study Group Brain Tumour Committee. J. Clin. Oncol. *23*, 7951–7957.

Ellison, D.W., Kocak, M., Dalton, J., Megahed, H., Lusher, M.E., Ryan, S.L., Zhao, W., Nicholson, S.L., Taylor, R.E., Bailey, S., and Clifford, S.C. (2011). Definition of disease-risk stratification groups in childhood medulloblastoma using combined clinical, pathologic, and molecular variables. J. Clin. Oncol. *29*, 1400–1407.

Frank, A.J., Hernan, R., Hollander, A., Lindsey, J.C., Lusher, M.E., Fuller, C.E., Clifford, S.C., and Gilbertson, R.J. (2004). The TP53-ARF tumor suppressor pathway is frequently disrupted in large/cell anaplastic medulloblastoma. Brain Res. Mol. Brain Res. *121*, 137–140.

Gustafson, W.C., Meyerowitz, J.G., Nekritz, E.A., Chen, J., Benes, C., Charron, E., Simonds, E.F., Seeger, R., Matthay, K.K., Hertz, N.T., et al. (2014). Drugging MYCN through an allosteric transition in Aurora kinase A. Cancer Cell *26*, 414–427.

Hovestadt, V., Remke, M., Kool, M., Pietsch, T., Northcott, P.A., Fischer, R., Cavalli, F.M., Ramaswamy, V., Zapatka, M., Reifenberger, G., et al. (2013). Robust molecular subgrouping and copy-number profiling of medulloblastoma from small amounts of archival tumour material using high-density DNA methylation arrays. Acta Neuropathol. *125*, 913–916.

Jones, D.T., Jäger, N., Kool, M., Zichner, T., Hutter, B., Sultan, M., Cho, Y.J., Pugh, T.J., Hovestadt, V., Stütz, A.M., et al. (2012). Dissecting the genomic complexity underlying medulloblastoma. Nature *488*, 100–105.

Kawauchi, D., Robinson, G., Uziel, T., Gibson, P., Rehg, J., Gao, C., Finkelstein, D., Qu, C., Pounds, S., Ellison, D.W., et al. (2012). A mouse model of the most aggressive subgroup of human medulloblastoma. Cancer Cell *21*, 168–180.

Kool, M., Korshunov, A., Remke, M., Jones, D.T., Schlanstein, M., Northcott, P.A., Cho, Y.J., Koster, J., Schouten-van Meeteren, A., van Vuurden, D., et al. (2012). Molecular subgroups of medulloblastoma: an international meta-analysis of transcriptome, genetic aberrations, and clinical data of WNT, SHH, Group 3, and Group 4 medulloblastomas. Acta Neuropathol. *123*, 473–484.

Lamont, J.M., McManamy, C.S., Pearson, A.D., Clifford, S.C., and Ellison, D.W. (2004). Combined histopathological and molecular cytogenetic stratification of medulloblastoma patients. Clin. Cancer Res. *10*, 5482–5493.

(B) Kaplan-Meier survival for GTML/*Trp53*$^{KI/KI}$ mice treated with MLN8237 (n = 10), GDC-0449 SHH antagonist (n = 4), or vehicle (n = 11) as indicated. (p, log rank test.)
(C) Longitudinal MRI analysis of tumor volume (n = 4) on the axial plane (top). Representative MRIs of the axial plane of MLN8237-treated animals compared to vehicle as indicated at day 0 and last day of treatment (bottom).
(D) H&E and immunohistochemical staining indicating levels of MYCN protein, cell proliferation (Ki-67), apoptosis (cleaved caspase 3), or mitotic activity as measured by phosphorylated Ser10 on histone H3 (H3 p-S10) after treatment with GDC-0449 or MLN8237. Scale bar, 50 μm.
(E) Immunoblotting of MYCN protein levels, and total and phosphorylated Thr288 on Aurora-A (p-T288 Aurora-A) in MLN8237-treated tumor tissues. For (D) and (E), animals were treated with vehicle, GDC-0449, or MLN8237 for 48 hr, and samples were taken 2 hr after final administration of agent.
Error bars represent mean ± SD. See also Figure S4.

Langdon, J.A., Lamont, J.M., Scott, D.K., Dyer, S., Prebble, E., Bown, N., Grundy, R.G., Ellison, D.W., and Clifford, S.C. (2006). Combined genome-wide allelotyping and copy number analysis identify frequent genetic losses without copy number reduction in medulloblastoma. Genes Chromosomes Cancer *45*, 47–60.

Lannering, B., Rutkowski, S., Doz, F., Pizer, B., Gustafsson, G., Navajas, A., Massimino, M., Reddingius, R., Benesch, M., Carrie, C., et al. (2012). Hyperfractionated versus conventional radiotherapy followed by chemotherapy in standard-risk medulloblastoma: results from the randomized multicenter HIT-SIOP PNET 4 trial. J. Clin. Oncol. *30*, 3187–3193.

Louis, D.N., Ohgaki, H., Wiestler, O.D., Cavenee, W.K., Burger, P.C., Jouvet, A., Scheithauer, B.W., and Kleihues, P. (2007). The 2007 WHO classification of tumours of the central nervous system. Acta Neuropathol. *114*, 97–109.

McManamy, C.S., Pears, J., Weston, C.L., Hanzely, Z., Ironside, J.W., Taylor, R.E., Grundy, R.G., Clifford, S.C., and Ellison, D.W. (2007). Nodule formation and desmoplasia in medulloblastomas-defining the nodular/desmoplastic variant and its biological behavior. Brain Pathol. *17*, 151–164.

Nicholson, J., Wickramasinghe, C., Ross, F., Crolla, J., and Ellison, D. (2000). Imbalances of chromosome 17 in medulloblastomas determined by comparative genomic hybridisation and fluorescence in situ hybridisation. Mol. Pathol. *53*, 313–319.

Northcott, P.A., Jones, D.T., Kool, M., Robinson, G.W., Gilbertson, R.J., Cho, Y.J., Pomeroy, S.L., Korshunov, A., Lichter, P., Taylor, M.D., and Pfister, S.M. (2012a). Medulloblastomics: the end of the beginning. Nat. Rev. Cancer *12*, 818–834.

Northcott, P.A., Shih, D.J., Peacock, J., Garzia, L., Morrissy, A.S., Zichner, T., Stütz, A.M., Korshunov, A., Reimand, J., Schumacher, S.E., et al. (2012b). Subgroup-specific structural variation across 1,000 medulloblastoma genomes. Nature *488*, 49–56.

Pei, Y., Moore, C.E., Wang, J., Tewari, A.K., Eroshkin, A., Cho, Y.J., Witt, H., Korshunov, A., Read, T.A., Sun, J.L., et al. (2012). An animal model of MYC-driven medulloblastoma. Cancer Cell *21*, 155–167.

Pfaff, E., Remke, M., Sturm, D., Benner, A., Witt, H., Milde, T., von Bueren, A.O., Wittmann, A., Schöttler, A., Jorch, N., et al. (2010). TP53 mutation is frequently associated with CTNNB1 mutation or MYCN amplification and is compatible with long-term survival in medulloblastoma. J. Clin. Oncol. *28*, 5188–5196.

Pfister, S., Remke, M., Benner, A., Mendrzyk, F., Toedt, G., Felsberg, J., Wittmann, A., Devens, F., Gerber, N.U., Joos, S., et al. (2009). Outcome prediction in pediatric medulloblastoma based on DNA copy-number aberrations of chromosomes 6q and 17q and the MYC and MYCN loci. J. Clin. Oncol. *27*, 1627–1636.

Pizer, B.L., and Clifford, S.C. (2009). The potential impact of tumour biology on improved clinical practice for medulloblastoma: progress towards biologically driven clinical trials. Br. J. Neurosurg. *23*, 364–375.

Pizer, B., Donachie, P.H., Robinson, K., Taylor, R.E., Michalski, A., Punt, J., Ellison, D.W., and Picton, S. (2011). Treatment of recurrent central nervous system primitive neuroectodermal tumours in children and adolescents: results of a Children's Cancer and Leukaemia Group study. Eur. J. Cancer *47*, 1389–1397.

Ramaswamy, V., Remke, M., Bouffet, E., Faria, C.C., Perreault, S., Cho, Y.J., Shih, D.J., Luu, B., Dubuc, A.M., Northcott, P.A., et al. (2013). Recurrence patterns across medulloblastoma subgroups: an integrated clinical and molecular analysis. Lancet Oncol. *14*, 1200–1207.

Randerson-Moor, J.A., Harland, M., Williams, S., Cuthbert-Heavens, D., Sheridan, E., Aveyard, J., Sibley, K., Whitaker, L., Knowles, M., Bishop, J.N., and Bishop, D.T. (2001). A germline deletion of p14(ARF) but not CDKN2A in a melanoma-neural system tumour syndrome family. Hum. Mol. Genet. *10*, 55–62.

Robinson, G., Parker, M., Kranenburg, T.A., Lu, C., Chen, X., Ding, L., Phoenix, T.N., Hedlund, E., Wei, L., Zhu, X., et al. (2012). Novel mutations target distinct subgroups of medulloblastoma. Nature *488*, 43–48.

Rudin, C.M., Hann, C.L., Laterra, J., Yauch, R.L., Callahan, C.A., Fu, L., Holcomb, T., Stinson, J., Gould, S.E., Coleman, B., et al. (2009). Treatment of medulloblastoma with hedgehog pathway inhibitor GDC-0449. N. Engl. J. Med. *361*, 1173–1178.

Rutkowski, S., Gerber, N.U., von Hoff, K., Gnekow, A., Bode, U., Graf, N., Berthold, F., Henze, G., Wolff, J.E., Warmuth-Metz, M., et al. (2009). Treatment of early childhood medulloblastoma by postoperative chemotherapy and deferred radiotherapy. Neuro-oncol. *11*, 201–210.

Ryan, S.L., Schwalbe, E.C., Cole, M., Lu, Y., Lusher, M.E., Megahed, H., O'Toole, K., Nicholson, S.L., Bognar, L., Garami, M., et al. (2012). MYC family amplification and clinical risk-factors interact to predict an extremely poor prognosis in childhood medulloblastoma. Acta Neuropathol. *123*, 501–513.

Schwalbe, E.C., Williamson, D., Lindsey, J.C., Hamilton, D., Ryan, S.L., Megahed, H., Garami, M., Hauser, P., Dembowska-Baginska, B., Perek, D., et al. (2013). DNA methylation profiling of medulloblastoma allows robust subclassification and improved outcome prediction using formalin-fixed biopsies. Acta Neuropathol. *125*, 359–371.

Shakhova, O., Leung, C., van Montfort, E., Berns, A., and Marino, S. (2006). Lack of Rb and p53 delays cerebellar development and predisposes to large cell anaplastic medulloblastoma through amplification of N-Myc and Ptch2. Cancer Res. *66*, 5190–5200.

Shih, D.J., Northcott, P.A., Remke, M., Korshunov, A., Ramaswamy, V., Kool, M., Luu, B., Yao, Y., Wang, X., Dubuc, A.M., et al. (2014). Cytogenetic prognostication within medulloblastoma subgroups. J. Clin. Oncol. *32*, 886–896.

Sturm, D., Witt, H., Hovestadt, V., Khuong-Quang, D.A., Jones, D.T., Konermann, C., Pfaff, E., Tönjes, M., Sill, M., Bender, S., et al. (2012). Hotspot mutations in H3F3A and IDH1 define distinct epigenetic and biological subgroups of glioblastoma. Cancer Cell *22*, 425–437.

Swartling, F.J., Grimmer, M.R., Hackett, C.S., Northcott, P.A., Fan, Q.W., Goldenberg, D.D., Lau, J., Masic, S., Nguyen, K., Yakovenko, S., et al. (2010). Pleiotropic role for MYCN in medulloblastoma. Genes Dev. *24*, 1059–1072.

Tabori, U., Baskin, B., Shago, M., Alon, N., Taylor, M.D., Ray, P.N., Bouffet, E., Malkin, D., and Hawkins, C. (2010). Universal poor survival in children with medulloblastoma harboring somatic TP53 mutations. J. Clin. Oncol. *28*, 1345–1350.

Taketo, M., Schroeder, A.C., Mobraaten, L.E., Gunning, K.B., Hanten, G., Fox, R.R., Roderick, T.H., Stewart, C.L., Lilly, F., Hansen, C.T., et al. (1991). FVB/N: an inbred mouse strain preferable for transgenic analyses. Proc. Natl. Acad. Sci. USA *88*, 2065–2069.

Tamayo, P., Scanfeld, D., Ebert, B.L., Gillette, M.A., Roberts, C.W., and Mesirov, J.P. (2007). Metagene projection for cross-platform, cross-species characterization of global transcriptional states. Proc. Natl. Acad. Sci. USA *104*, 5959–5964.

Taylor, M.D., Northcott, P.A., Korshunov, A., Remke, M., Cho, Y.J., Clifford, S.C., Eberhart, C.G., Parsons, D.W., Rutkowski, S., Gajjar, A., et al. (2012). Molecular subgroups of medulloblastoma: the current consensus. Acta Neuropathol. *123*, 465–472.

Van Maerken, T., Rihani, A., Van Goethem, A., De Paepe, A., Speleman, F., and Vandesompele, J. (2014). Pharmacologic activation of wild-type p53 by nutlin therapy in childhood cancer. Cancer Lett. *344*, 157–165.

Workman, P., Aboagye, E.O., Balkwill, F., Balmain, A., Bruder, G., Chaplin, D.J., Double, J.A., Everitt, J., Farningham, D.A.H., Glennie, M.J., et al.; Committee of the National Cancer Research Institute (2010). Guidelines for the welfare and use of animals in cancer research. Br. J. Cancer *102*, 1555–1577.

Zhukova, N., Ramaswamy, V., Remke, M., Pfaff, E., Shih, D.J., Martin, D.C., Castelo-Branco, P., Baskin, B., Ray, P.N., Bouffet, E., et al. (2013). Subgroup-specific prognostic implications of TP53 mutation in medulloblastoma. J. Clin. Oncol. *31*, 2927–2935.

Cancer Cell, Volume *27*

Supplemental Information

Combined MYC and P53 Defects Emerge at Medulloblastoma Relapse and Define Rapidly Progressive, Therapeutically Targetable Disease

Rebecca M. Hill, Sanne Kuijper, Janet C. Lindsey, Kevin Petrie, Ed C. Schwalbe, Karen Barker, Jessica K.R. Boult, Daniel Williamson, Zai Ahmad, Albert Hallsworth, Sarra L. Ryan, Evon Poon, Simon P. Robinson, Ruth Ruddle, Florence I. Raynaud, Louise Howell, Colin Kwok, Abhijit Joshi, Sarah Leigh Nicholson, Stephen Crosier, David W. Ellison, Stephen B. Wharton, Keith Robson, Antony Michalski, Darren Hargrave, Thomas S. Jacques, Barry Pizer, Simon Bailey, Fredrik J. Swartling, William A. Weiss, Louis Chesler, and Steven C. Clifford

SUPPLEMENTAL DATA

Table S1 (related to Table1). Molecular details of *CTNNB1* and *TP53* mutated medulloblastomas

Patient number	Mutated gene	Protein	p53 database[1]	Acquired mutation	Molecular subgroup
13	*CTNNB1*	Ser34Phe	n/a	No	MB_{WNT}
14	*CTNNB1*	Ser37deletion	n/a	Unknown	MB_{WNT}
5	*TP53*	Gly245Val	Yes	Yes	MB_{SHH}
8	*TP53*	Arg282Trp	Yes	No	MB_{SHH}
10	*TP53*	Arg282Trp	Yes	No	MB_{SHH}
12	*TP53*	Arg273His	Yes	No	MB_{SHH}
13	*TP53*	Arg158Cys and Arg282Trp	Yes	No	MB_{WNT}
14	*TP53*	Arg273His	Yes	Unknown	MB_{WNT}
22	*TP53*	Pro152Leu	Yes	Yes	MB_{Group4}
29	*TP53*	Arg175His	Yes	Unknown	Unknown

[1](Soussi et al. 2006) Ser, Serine; Phe, phenylalanine; Arg, Arginine; Cys, Cysteine; Trp, Tryptophan; His, Histidine; Gly, Glycine; Val, Valine; Pro, Proline; Leu, Leucine. n/a, not applicable. *CTNNB1* encodes Beta-catenin.

Table S2 (related to Table1). Subgroup distribution in the present paired study cohort of relapsed medulloblastomas compared to previously published data

Independent published cohort of medulloblastomas sampled at relapse[1] (Ramaswamy et al., 2013)

Molecular subgroup	Paired relapse study	Independent published study	
	Relapse	Relapse	p value
MB_{SHH}	12/25 (48%)	21/51 (41%)	0.6275
MB_{WNT}	2/25 (8%)	1/51 (2%)	0.2504
MB_{Group3}	2/25 (8%)	9/51 (18%)	0.3215
MB_{Group4}	9/25 (36%)	20/51 (39%)	1

Relapsing tumours in an unbiased trial-based cohort of medulloblastomas sampled at diagnosis[2] (Schwalbe et al., 2013)

Molecular subgroup	Paired relapse study	Independently published study	
	Relapse	Relapsing tumours (sampled at dignosis)	p value
MB_{SHH}	5/18 (28%)	17/64 (27%)	1
MB_{WNT}	2/18 (11%)	1/64 (2%)	0.1198
MB_{Group3}	2/18 (11%)	16/64 (25%)	0.3349
MB_{Group4}	9/18 (50%)	30/64 (47%)	1

[1]Based on all patients with available subgroup data.
[2]Based on all patients receiving radiotherapy aged 3-16 years. p, Fisher's exact test.

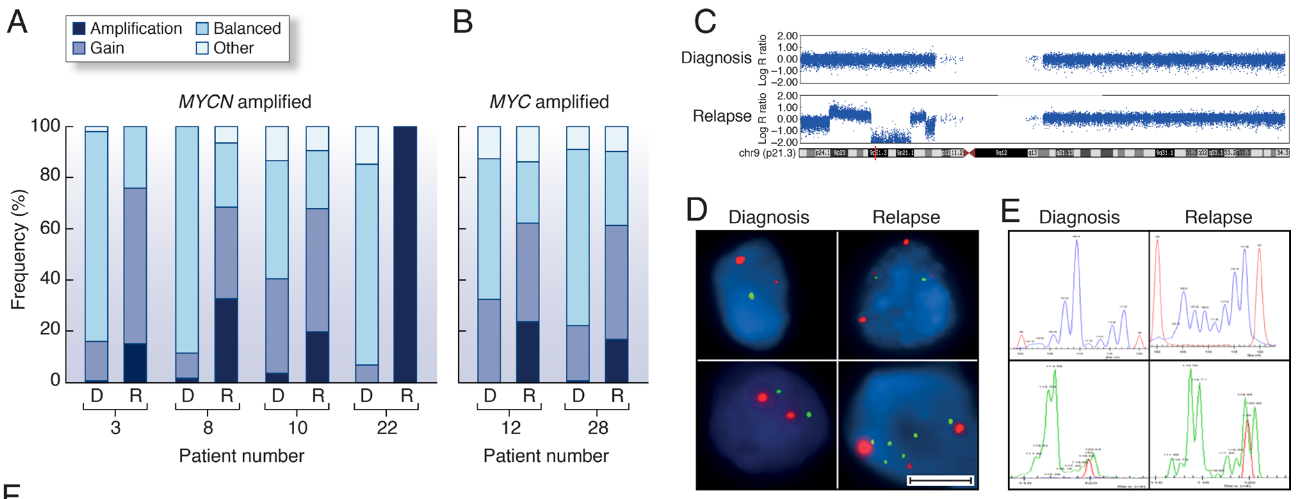

F

Treatment group	Overall survival
Non-infant: RT at diagnosis	0/22 (0%)[1]
Non-infant: RT at relapse	0/1 (0%)
Infant: No RT	0/2 (0%)
Infant: RT at relapse	4/4 (100%)

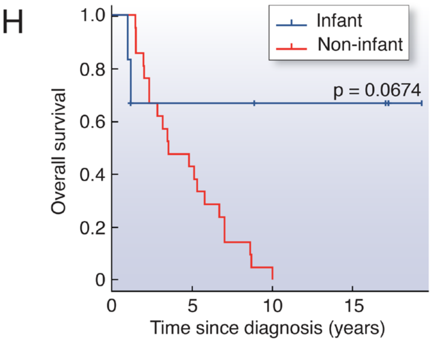

G

Variable		Number of cases n = 29	Univariate HR	Univariate 95% CI	Univariate p value	Multivariate p value
Age	Infant	6/29 (21%)	0.171	0.04 – 0.77	**0.021**	**0.021**
	Non-infant	23/29 (79%)				
Gender	Male	18/29 (62%)	0.385	0.16 – 0.92	**0.032**	ns
	Female	11/29 (38%)				
RT at diagnosis	Yes	22/29 (76%)	3.715	1.05 – 13.21	**0.043**	ns
	No	7/29 (24%)				
Resection	STR	9/29 (31%)	2.281	0.9 – 5.79	0.083	ns
	GTR	20/29 (69%)				
Pathology	LCA	3/26 (12%)	1.758	0.49 – 6.33	0.388	ns
	Non-LCA	23/26 (88%)				
Metastatic stage	M+	10/29 (34%)	0.886	0.37 – 2.10	0.784	ns
	M–	19/29 (66%)				

Figure S1 (related to Figure 1). Analysis of enrichment and acquisition of molecular defects at relapse and patient survival following medulloblastoma recurrence

(A) Frequency as determined by iFISH of intra-tumoral *MYCN* copy number defects at diagnosis (D) and relapse (R).

(B) Frequency as determined by iFISH of intra-tumoral *MYC* copy number defects at diagnosis (D) and relapse (R).

(C) *CDKN2A* ($p14^{ARF}$) homozygous deletion at relapse (Illumina Omni-express SNP array; *CDKN2A* position, red).

(D) Evolution of chromosome 17 defects and polyploidy by iFISH. (top left) 17p loss (green versus centromeric control (red)), (bottom left) balanced 17q, (top right) maintenance of 17p loss with development of polyploidy, (bottom right) acquisition of polyploidy and 17q gain (overall i(17q)). Scale bar indicates 5 µM.

(E) Acquisition of microsatellite instability at relapse (diagnosis; normal heterozygous profiles and relapse; multiple peaks demonstrating instability).

(F) Survival status of 29 relapsed medulloblastoma patients by treatment received. Infant, < 4.0 years at diagnosis; RT, radiotherapy. [1]Two patients died from treatment complications.

(G) Unadjusted Cox proportional hazard models assessing the prognostic significance (overall survival) of clinico-pathological variables at diagnosis in relapsing patients. STR, subtotal resection; GTR, gross total resection; LCA, large-cell/anaplastic; M+, M2+ disease; M-, M0/M1.

(H) Kaplan-Meier curve (whole cohort) showing survival of infant patients following recurrence (p, log-rank test, Bonferroni corrected).

Table S3 (related to Figure 1). Incidence of clinical, pathological and molecular features in the present relapsed study cohort (at diagnosis and relapse) compared to large historic cohorts

Clinicopathological and molecular features		Historic studies[1]	Paired relapse study			
		Diagnosis	Diagnosis	p value	Relapse	p value
Gender	Male	585/952 (61%)	18/29 (62%)		18/29 (62%)	
	Female	367/952 (39%)	11/29 (38%)	1	11/29 (38%)	1
	Male:female ratio	1.6:1	1.6:1		1.6:1	
Age	Age range in years	0.3–52	0.1–33.7			
	Infants (<4 years)	167/943 (17%)	6/29 (21%)	0.6261*		
	Children (4–16 years)	599/943 (64%)	22/29 (76%)			
	Adults (>16 years)	177/943 (19%)	1/29 (3%)			
Pathology	CLA	938/1277 (74%)	16/26 (61%)		14/24 (58%)	
	DN	183/1277 (14%)	7/26 (27%)		4/24 (17%)	
	LCA	156/1277 (12%)	3/26 (12%)	1*	6/24 (25%)	0.1073*
Metastatic stage	M–	608/834 (73%)	9/22 (41%)	**0.0026**	6/24 (25%)	**<0.0001**
	M+	226/834 (27%)	13/22 (59%)		18/24 (75%)	
Resection	GTR	267/317 (84%)	20/29 (69%)	0.0657	n/a	
	STR	50/317 (16%)	9/29 (31%)		n/a	
Molecular subgroup	MB_{SHH}	153/550 (28%)	12/25 (48%)	**0.0402***	12/25 (48%)	**0.0402***
	MB_{WNT}	60/550 (11%)	2/25 (8%)	1	2/25 (8%)	1
	MB_{Group3}	149/550 (27%)	2/25 (8%)	**0.0355***	2/25 (8%)	**0.0355***
	MB_{Group4}	188/550 (34%)	9/25 (36%)	0.8324	9/25 (36%)	0.8324
Molecular defects	*MYC/MYCN* amplification	50/552 (9%)	2/25 (8%)	1	9/29 (31%)	**0.0012**
	MYC amplification	17/552 (3%)	1/25 (4%)	0.5549	4/29 (14%)	**0.0166**
	MYCN amplification	33/552 (6%)	1/25 (4%)	1	5/29 (17%)	**0.034**
	TP53 mutations	21/310 (7%)	4/26 (15%)	0.1157	8/29 (28%)	**0.0013**
	Chromosome 17 defects	138/260 (53%)	8/21 (38%)	0.2562	10/21 (48%)	0.6562
Combined molecular defects	*TP53* mutation and *MYC/MYCN* amplification	8/310 (3%)	0/26 (0%)	1	6/29 (21%)	**0.0004**
	TP53 mutation and *MYC* amplification	1/310 (<1%)	0/26 (0%)	1	2/29 (7%)	**0.0201**
	TP53 mutation and *MYCN* amplification	7/310 (2%)	0/26 (0%)	1	4/29 (14%)	**0.0094**

[1]Historic studies: (Kool et al,. 2012; Pfister et al,. 2009; Ryan et al,. 2012; McManamy et al,. 2007; Lannering et al,. 2012; Pfaff et al,. 2010). Pathology variant: CLA, classic; LCA, large-cell/anaplastic; DN, desmoplastic/nodular. Metastatic stage: M-, M0; M+, M1-4. Resection: GTR, gross total resection; STR sub-total resection. Data are shown as a proportion and percentage of all data available. *Patients with each specific feature compared against all others. Features significantly enriched at diagnosis and relapse are in bold (p, Fisher's exact test). n/a, not applicable.

A

Correlative analysis at diagnosis

	P53 defect	Ch17 defects	LCA pathology	Gender	Resection	Metastatic disease	Ploidy	MB_{Group4}	MB_{SHH}	*MYC* amplification
Ch17 defects	0.57(1)									
LCA pathology	0.53(1)	0.57(1)								
Gender	1(1)	1(1)	0.22(1)							
Resection	1(1)	1(1)	1(1)	1(1)						
Metastatic disease	0.25(1)	1(1)	1(1)	0.66(1)	0.38(1)					
Ploidy	1(1)	1(1)	1(1)	1(1)	0.59(1)	1(1)				
MB_{Group4}	0.08(1)	0.57(1)	1(1)	0.33(1)	0.62(1)	0.62(1)	1(1)			
MB_{SHH}	0.06(1)	1(1)	1(1)	0.33(1)	1(1)	1(1)	1(1)			
MYC amplification	*MYC* amplification not detected at diagnosis									
MYCN amplification	1(1)		1(1)	1(1)	0.39(1)	1(1)		1(1)	1(1)	

Correlative analysis at relapse

	P53 defect	Ch17 defects	LCA pathology	Gender	Resection	Metastatic disease	Ploidy	MB_{Group4}	MB_{SHH}	*MYC* amplification	*MYCN* amplification
Ch17 defects	0.57(1)										
LCA pathology	**0.03**(1)	1(1)									
Gender	0.39(1)	1(1)	0.28(1)								
Resection											
Metastatic disease	0.37(1)	1(1)	0.28(1)	0.17(1)							
Ploidy	0.37(1)	0.62(1)	1(1)	0.37(1)		1(1)					
MB_{Group4}	0.05(1)	1(1)	0.58(1)	0.33(1)		0.31(1)	0.28(1)				
MB_{SHH}	0.33(1)	0.22(1)	1(1)	0.33(1)		0.09(1)	0.3(1)				
MYC amplification	0.12(1)	1(1)	0.05(1)	0.12(1)		0.09(1)	0.59(1)	0.21(1)	1(1)		
MYCN amplification	**0.01**(0.49)	1(1)	0.51(1)	1(1)		1(1)	0.59(1)	1(1)	0.17(1)		
MYC/MYCN amplification	**0.0004(0.02)**	1(1)	**0.03**(1)	0.39(1)		0.17(1)	0.19(1)	0.13(1)	0.27(1)		

B

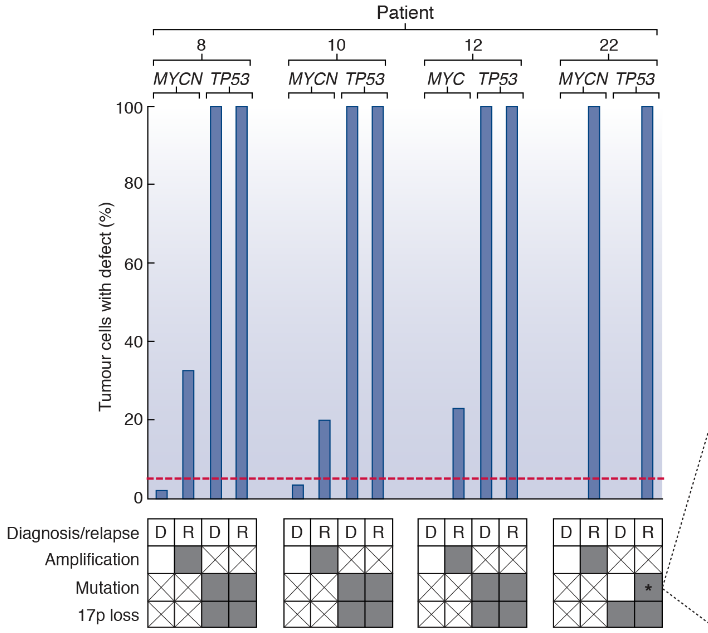

Next-generation sequencing		
**Results**	**Diagnosis**	**Relapse**
Number of reads	1917	1578
Wild-type *TP53* (CCG)	100%	12%
Mutant *TP53* (CTG)	0%	88%

Figure S2 (related to Figure 2). Analysis of combined P53 pathway defects and *MYC/MYCN* amplification at relapse

(A) *MYC/MYCN* amplification and P53 pathway defects at relapse are significantly correlated. Correlative p values (p, Fisher's exact test) at diagnosis and relapse are shown for patients receiving standard upfront radiotherapy and chemotherapy before and after (in parentheses) adjustment for multiple testing (Bonferroni correction). Cross-hatched boxes, not relevant.

(B) Assessment of intra-tumoral molecular heterogeneity supports the development of combined *MYC* family gene amplification and *TP53* mutation by both clonal enrichment and de novo acquisition. Estimated percentages of *MYC/MYCN*-amplified (by iFISH; >5% cells, dashed red line) and *TP53* mutated (estimated peak heights, mutant versus wild-type) tumor cells at diagnosis (D) and relapse (R) in four patients with available material support clonal enrichment (e.g. *MYCN* amplification in patients 8 & 10) and de novo acquisition of combined defects at relapse (e.g. *MYC* amplification in patient 12; *MYCN* amplification and *TP53* mutation in patient 22). Patient 22 showed no evidence of *TP53* mutation at diagnosis, based on >1900 next-generation sequencing reads (*expanded box). All *TP53* mutated tumors also displayed chromosome 17p loss. (bottom grid) grey, present; white, absent; cross-hatched, not relevant.

Table S4 (related to Figure 2). Combined *MYC/MYCN* amplification and P53 pathway defects predict a significantly shorter time to death following relapse

Variable		Number of cases	Univariate			Multivariate
			HR	95% CI	p value	p value
P53 pathway defect & *MYC/MYCN* amplification at relapse	Yes<br>No	7/20 (35%)<br>13/20 (65%)	8.185	1.93–34.19	**0.004**	**0.004**
MYC/MYCN amplification at relapse	Yes<br>No	7/20 (35%)<br>13/20 (65%)	8.185	1.93–34.19	**0.004**	ns
P53 pathway defect at relapse	Yes<br>No	8/20 (40%)<br>12/20 (60%)	3.692	1.26–10.78	**0.017**	ns
MYCN amplification at relapse	Yes<br>No	4/20 (20%)<br>16/20 (80%)	5.633	1.35–23.44	**0.017**	ns
Pathology at diagnosis	LCA<br>Non-LCA	3/17 (18%)<br>14/17 (82%)	6.304	1.22–32.45	**0.028**	ns
P53 pathway defect at diagnosis	Yes<br>No	4/17 (24%)<br>13/17 (76%)	2.919	0.86–9.91	0.086	ns
Ch17 defects at relapse	Yes<br>No	8/14 (57%)<br>6/14 (43%)	3.149	0.78–12.71	0.107	ns
MYC amplification at relapse	Yes<br>No	3/20 (15%)<br>17/20 (85%)	2.905	0.74–11.39	0.126	ns
Subgroup	MB_{SHH}<br>MB_{Group4}	5/13 (38%)<br>8/13 (62%)	2.272	0.67–7.67	0.186	ns
Ch17 defects at diagnosis	Yes<br>No	7/14 (50%)<br>7/14 (50%)	2.207	0.68–7.15	0.187	ns
Pathology at relapse	LCA<br>Non-LCA	5/17 (29%)<br>12/17 (71%)	1.613	0.53–4.88	0.397	ns
Metastatic stage at diagnosis	M+<br>M–	8/20 (40%)<br>12/20 (60%)	1.45	0.57–3.68	0.439	ns
Gender	Male<br>Female	12/20 (60%)<br>8/20 (40%)	0.728	0.29–1.86	0.506	ns
Ploidy at relapse	Yes<br>No	8/17 (47%)<br>9/17 (53%)	0.835	0.31–2.25	0.722	ns
Metastatic stage at relapse	M+<br>M–	10/18 (56%)<br>8/18 (44%)	0.857	0.33–2.26	0.754	ns
Ploidy at diagnosis	Yes<br>No	6/15 (40%)<br>9/15 (60%)	1.03	0.36–2.99	0.956	ns
Resection at diagnosis	STR<br>GTR	5/20 (25%)<br>15/20 (75%)	0.994	0.32–3.07	0.992	ns

Univariate and multivariate Cox proportional hazard models are shown assessing the relationship between time from relapse to death and clinical, pathological and molecular disease features, in patients receiving conventional upfront therapy. HR, hazard ratio; CI, confidence interval; LCA, large-cell/anaplastic; Ch17, chromosome 17; M+, M2+; M-, M0/M1; STR, subtotal resection; GTR, gross total resection; ns, not significant. *MYC/MYCN* amplification at diagnosis and microsatellite instability at relapse were not assessed (only one case observed).

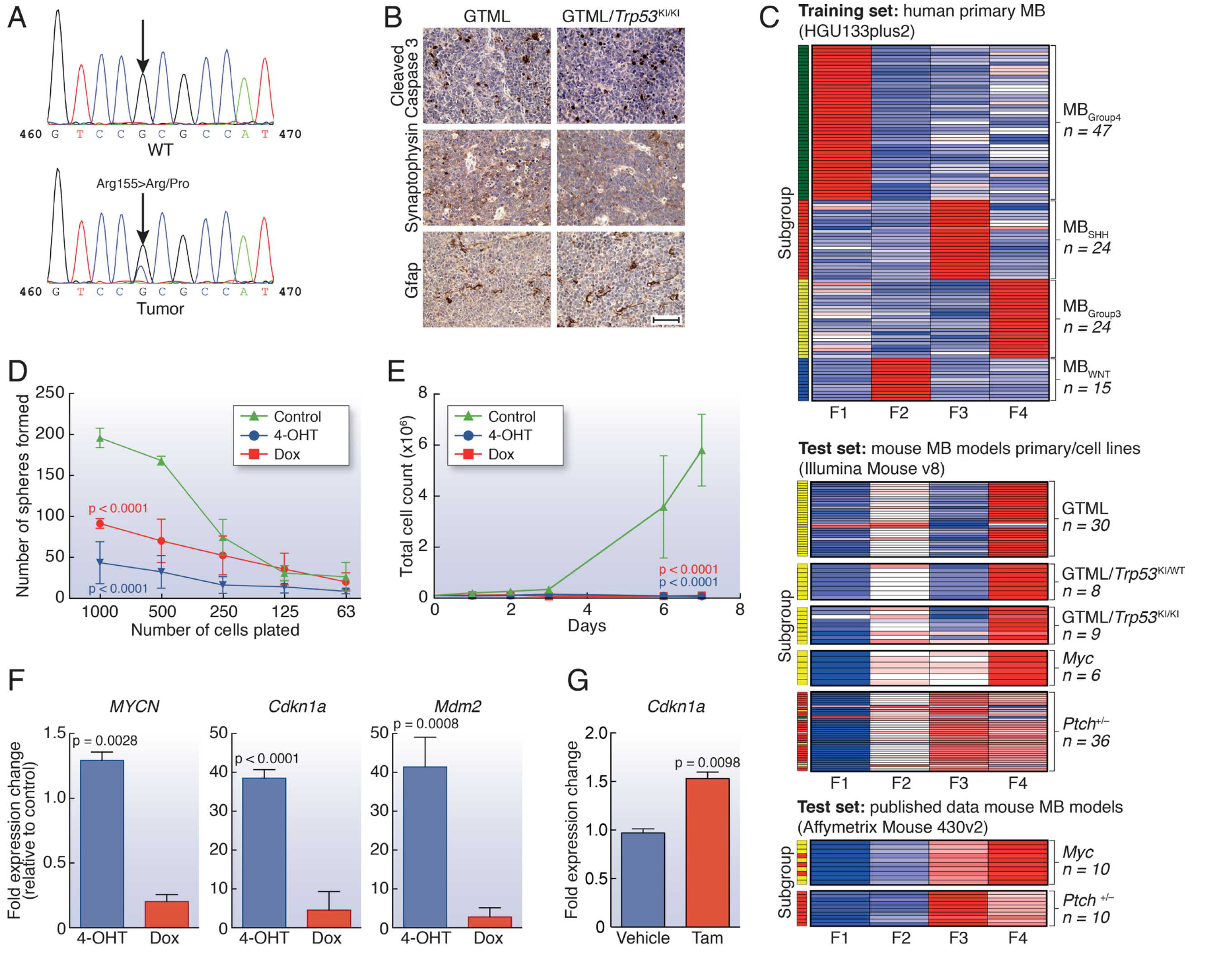

Figure S3 (related to Figure 3). Characterization of GTML/*Trp53*$^{KI/KI}$ and GTML mice

(A) Representative sequence indicates mutation in exon 5 (bottom panel), codon Arg155 leading to an amino acid change from arginine (CGC) to proline (CCC) in the tumor and not in the remaining brain tissue (top panel).

(B) H&E and immunohistochemical staining indicating apoptosis (Cleaved caspase 3), and levels of GFAP (a glial marker) and synaptophysin (a marker for neuroendocrine tumors) in GTML/*Trp53*$^{KI/KI}$ and GTML transgenic mice. Scale bar indicates 50 μm.

(C) Heatmap showing results of a classification experiment whereby human MB expression profiles were converted to 4 metagenes that describe each of the four MB sub-types using Non-negative Matrix Factorization (NMF). A Support Vector Machine (SVM) was used to create a classifier trained on the human metagenes and tested on the NMF metagenes projected from human onto mouse model expression profiles. Blue-red heatmap shows expression of subgroup specific NMF metagenes. Colored side bar shows sub-group classification calls (red, MB_{SHH}; blue, MB_{WNT}; yellow, MB_{Group3}; green, MB_{Group4}; grey, low confidence i.e. no call).

(D) Limiting dilution assay showing numbers of GTML/*Trp53*$^{KI/KI}$ neurospheres formed after treatment (3 days) with 4-OHT or dox compared to untreated control. p, two-way Anova.

(E) Limiting dilution assay showing cell growth after treatment (3 days) with 4-OHT or dox compared to untreated control. p, two-way Anova.

(F) Real-time quantitative PCR (Taqman) of human *MYCN* mRNA expression levels after dox treatment, and *Cdkn1a* and *Mdm2* mRNA expression levels after 4-OHT treatment. p, unpaired t test.

(G) Quantitative analysis of *Cdkn1a* mRNA expression in situ in tumors from GTML/*Trp53*$^{KI/KI}$ mice treated with vehicle or tamoxifen (Tam) as indicated (see Figure 3G). Expression was quantified using RNAscope SpotStudio and data are presented as relative change in the average number of spots per μm^2 of tumor cells analysed. p, unpaired t test.

Error bars represent mean ± SD.

Table S5 (related to Figure 3). *Trp53* mutations in GTML mice

		Sequence variant		Somatic *TP53* mutation count in human cancer[1]
Mouse ID#	Tissue	cDNA	Protein	
**10519**	**Tumor**	**734G>GA**	**Arg245Arg/His**	**1544 (Arg248)[2]**
10519	Brain	None detected	Wild type	n/a
**10087**	**Tumor**	**404C>CT**	**Ala135Ala/Val**	**110 (Ala138)**
10087	Brain	None detected	Wild type	n/a
**26826**	**Tumor**	**464G>GC**	**Arg155Arg/Pro**	**264 (Arg158)**
26826	Brain	None detected	Wild type	n/a
**10933**	**Tumor**	**464G>GC**	**Arg155Arg/Pro**	**264 (Arg158)**
10933	Brain	None detected	Wild type	n/a
**14658**	**Tumor**	**701T>C**	**Met234Thr**	**214 (Met237)**
14658	Brain	None detected	Wild type	n/a
**11185**	**Tumor**	**839G>T, 841A>C**	**Arg280Leu, Thr281Pro**	**103 (Arg283), 30 (Thr284)**
**14634**	**Tumor**	**382_405 het_delAATAAGCTATTCTGCCAGCTGGCG**	Wild type	n/a
**10545**	**Tumor**	**577C>CG**	**Arg193Arg/Gly**	**230 (Arg196)**
**19780**	**Tumor**	**734G>A**	**Arg245His**	**1544 (Arg248)**
**2737**	**Tumor**	**526C>T**	**His176Tyr**	**341 (His179)**
**9303**	**Tumor**	None detected	Wild type	n/a
9303	Tail	None detected	Wild type	n/a
**11153**	**Tumor**	None detected	Wild type	n/a

[1](Soussi et al. 2006). [2]Equivalent human p53 amino acids are shown in parenthesis. Ala, Alanine; Arg, Arginine; Cys, Cysteine; Gly, Glycine; His, Histidine; Leu, Leucine; Met, Methionine; Pro, Proline; Thr, Threonine; Tyr, Tyrosine; Val, Valine. n/a, not applicable.

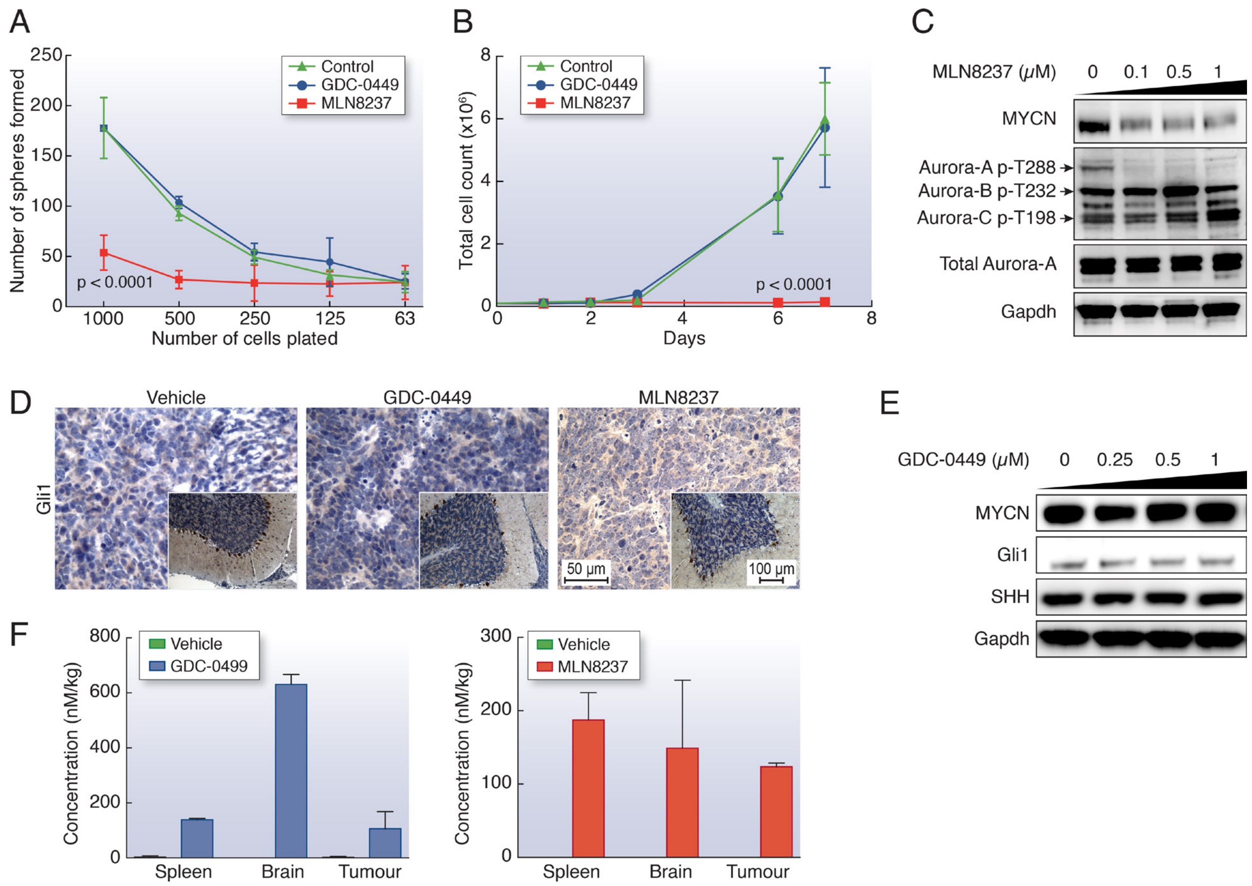

Figure S4 (related to Figure 4). Treatment with the Aurora-A kinase inhibitor MLN8237 inhibits medulloblastoma cell growth *in vitro* increases survival in GTML/*Trp53*$^{KI/KI}$ mice

(A) Limiting dilution assay showing numbers of GTML/*Trp53*$^{KI/KI}$ neurospheres formed after treatment (3 days) with GDC-0449 (blue) or MLN8237 (red) compared to untreated control (p, two-way Anova).

(B) Limiting dilution assay showing cell growth after treatment with GDC-0449 (blue) or MLN8237 (red) compared to untreated control (p, two-way Anova).

(C) Immunoblot analysis of MYCN, and phosphorylated Aurora-A (T288), Aurora-B (T232), Aurora-C (T198) and total Aurora-A protein levels in GTML/*Trp53*$^{KI/KI}$ neurospheres treated with MLN8237 at the indicated concentrations.

(D) Immunohistochemical analysis of Gli1 expression in MLN8237, GDC-0449 and vehicle treated tumor tissues. Scale bars represent 50 µm or 100 µm (inset) as indicated.

(E) Immunoblot analysis of MYCN, Gli1 and Sonic Hedgehog (SHH) protein levels in GTML/*Trp53*$^{KI/KI}$ neurospheres treated with GDC-0449 at the indicated concentrations.

(F) In vivo compound measurement of GDC-0449 and MLN8237 in spleen, brain and tumor tissue.

Error bars represent mean ± SD.

SUPPLEMENTAL EXPERIMENTAL PROCEDURES

Analysis of Molecular Subgroup Status by DNA Methylation Profiling

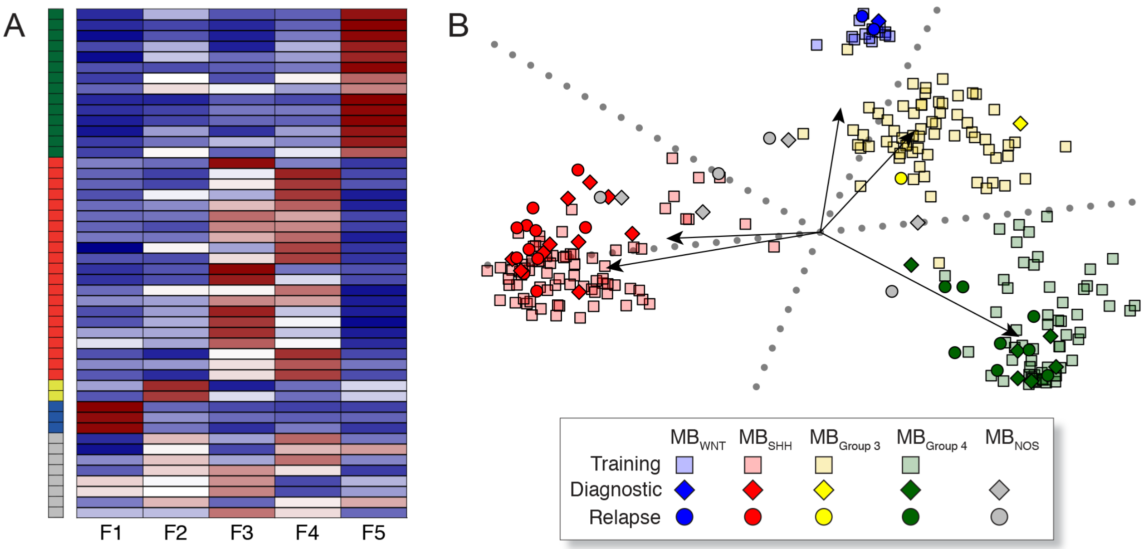

(A) Identification of subgroup membership using metagene patterns of the diagnostic and relapse samples within our study cohort. 5 metagenes (F1 to F5) were identified in the training cohort of 225 medulloblastomas using consensus NMF clustering; 2 metagenes (F3 and F4) identified the SHH subgroup, consistent with recent reports of heterogeneity within the SHH subgroup (Kool et al., 2014). Metagene projection (Tamayo et al., 2007) was employed to derive metagene values for the study cohort that were subsequently used to assign subgroup and assess classification confidence. Samples are shown in rows; samples confidently assigned to a subgroup are labelled by their assignation (red, MB_{SHH}; blue, MB_{WNT}; yellow, MB_{Group3}; green, MB_{Group4}). Samples within our study cohort that were unable to be confidently assigned (confidence score < 0.7) are labelled grey (MB_{NOS}). High, intermediate and low metagene values are shown red, white and blue, respectively.

(B) Comparison of metagene patterns for diagnostic and relapse samples within our study cohort, with metagene patterns from the training cohort. Bi-plot of principal component analysis of combined training, diagnostic and relapsing cohorts. Arrows show projections of five metagenes along first and second principal components. Individual cases are plotted by their principal component scores. Training cohort cases are shown as squares, diagnostic cohort cases as diamonds and relapsing cohort cases as circles. Additionally, cases are labelled by their assigned subgroup (red, MB_{SHH}; blue, MB_{WNT}; yellow, MB_{Group3}; green, MB_{Group4}. Cases with an unclear subgroup (confidence score < 0.7) are labelled grey (MB_{NOS})) and were not included in our subgroup analysis. Finally, training cohort cases are shown with transparency, to emphasise the members of our study cohort.

Analysis of *TP53* Status in Clinical Samples

TP53 status was assessed by direct polymerase chain reaction (PCR) based DNA sequence analysis of exons 4-9 using the following FAST PCR conditions: 95ºC for 40s; 35 cycles of 94ºC for 0s, then 64ºC for 15s; 72ºC for 45s (see below for primer sequences). Cases positive for P53 nuclear staining on immunohistochemistry (IHC) with no apparent mutation had an extended screen of exons 2-11 (see below for primer sequences). One tumor pair (patient 22, Figure S2B) that showed acquisition of a *TP53* mutation in exon 5 was assessed by next-generation sequencing using primers for exon 5 as previously described (Grossmann et al., 2011). Primer validation was performed according to manufacturer's instructions (Fluidigm) and PCR products checked using a 2100 BioAnalyzer (Agilent Technologies). Barcoded PCR products were pooled and run in a single sequencing experiment on a Roche 454 FLX sequencer (454 Life Sciences).

Primers for *TP53* PCR reaction

Location	Forward primer 5'–3'	Reverse primer 5'–3'
Exon 2	CCAGGGTTGGAAGCGTCTC	GACAAGAGCAGAAAGTCAGTCC
Exon 3/4	CATGGGACTGACTTTCTGCTC	CTTCATCTGGACCTGGGTCT
Exon 4 (part)	GGACGATATTGAACAATGGTT	ATGGAAGCCAGCCCCTCAG
Exon 4	GGCTGAGGACCTGGTCCTCTGA	GCCAGGCATTGAAGTCTCATGG
Exon 5	ATCTGTTCACTTGTGCCCTG	CAACCAGCCCTGTCGTCTCTC
Exon 6	GCCTCTGATTCCTCACTGAT	GGAGGGCCACTGACAACCA
Exon 7	AAGGCGCACTGGCCTCATCTT	CAGGGGTCAGAGGCAAGCAGA
Exon 8	GAGCCTGGTTTTTTAAATGG	TTTGGCTGGGGAGAGGAGCT
Exon 9	AGCGAGGTAAGCAAGCAGG	GCCCCAATTGCAGGTAAAACAG
Exon 10	CTTCTCCCCCTCCTCTGTTGC	GAAGGCAGGATGAGAATGGA
Exon 11	GGCACAGACCCTCTCACTCAT	TGCTTCTGACGCACACCTATT

Microsatellite Instability and Polyploidy in Clinical Samples

Microsatellite instability was assessed by a panel of markers on different chromosomes; d9s942, d9s1748 d5s346, d2s123, *MYCL*, d18s69, s10s197, *TP53*, d17s2196, d17s936, d17s969, d17s974, d17s786 and d17s1866. Tumors were classed as showing microsatellite instability if > 40% of markers analyzed were unstable compared to the diagnostic sample (Boland et al., 1998; Jung et al., 2004; Langdon et al., 2006). Polyploidy, was defined as a modal score > 2 at ≥ 2 centromeric loci examined by iFISH (chromosomes 2, 8 and 17).

Trp53 Mutational Analysis

Genomic DNA was extracted from cell lines, tumors and where available, normal brain tissue using QIAamp DNA Mini kit (Qiagen). PCR amplification of exons 5-9 was performed using primers detailed below. Products were sequenced with the original PCR primers using the BigDye Terminator Cycle Sequencing Kit and an ABI 3730 Genetic Analyzer (Applied Biosystems). Sequences were analyzed using Mutation Surveyor software (SoftGenetics).

Primers for *Trp53* PCR reaction

Location	Forward primer 5'–3'	Reverse primer 5'–3'
Exon 5/6	GATCGTTACTCGGCTTGTCC	AAGACGCACAAACCAAAACA
Exon 7	CTATAGCCAGCCATTCCCG	AGGCAGAAGCTGGGGAAG
Exon 8/9	TACACACAGTCAGGATGGGG	ATGCGAGAGACAGAGGCAAT

Real-time PCR

Total RNA was isolated from cells or tumor tissue using the miRNAeasy minikit (Qiagen) and cDNA prepared using Superscript II Reverse Transcriptase (Life Technologies). Quantitative PCR (QT-PCR) was performed in triplicate using Taqman Gene Expression mix (Life Technologies) and gene-specific primers for *Cdkn1a* (Mm04205640), *Mdm2* (Mm01233136), *MYCN* (Hs00232074) and *Actb* (Mm00607939) (Life Technologies). Relative expression was calculated according to the ΔΔCt relative quantification method against the average expression of control cells treated with ethanol or vehicle treated tumors.

In Situ Proximity Ligation Assay

Duolink in situ proximity ligation assay (PLA; Olink Bioscience) was performed on GTML/*Trp53*$^{KI/KI}$ neurospheres. Cells were fixed in 4% paraformaldehyde for 20 min, permeabilized with 0.5% Triton X-100, and blocked with 1% BSA for 30 min at room temperature followed by incubation with paired primary antibodies, MYCN (OP-13, Merck-Millipore) with Aurora-A (Genetex), overnight at 4ºC. PLA detection was performed as recommended by the manufacturer. Images were taken and analyzed using the Zeiss LSM700 confocal microscope and analyzed using DuoLink image analysis software.

Quantitative In Situ RNA Analysis

Dual color RNA *in situ* hybridization was performed using the RNAscope 2-plex Chromogenic Reagent Kit (Advanced Cell Diagnostics, ACD) according to the manufacturer's instructions. Paired double-Z oligonucleotide probes were designed against *Cdkn1a* using custom software as previously described (Wang et al., 2012). *Cdkn1a*-specific RNA target Z probe pairs (20) targeted bps 19 through 1240 of the *Cdkn1a* cDNA sequence (NM_007669.4). Probe sets specific for mouse *Ubc* (ubiquitin C), *Polr2a* (DNA-directed RNA polymerase II subunit RPB1) and *Ppib* (Peptidylprolyl Isomerase B, Cyclophilin B) and *dapB* (dihydrodipicolinate reductase) gene from *B. subtilis* were also used. FFPE tissue blocks were sectioned at 4 μm. Slides were baked for 1 hr at 60°C prior to use. After de-paraffinization and dehydration, the tissues were air dried and treated with peroxidase blocker before boiling at 100-104 °C in a pre-treatment solution for 15 min. Protease was then applied for 30 min at 40 °C. Target probes for each two-gene combination were premixed and hybridized together for 2 hr at 40 °C, followed by a series of signal amplification and washing steps. All hybridizations at 40 °C were performed in a HybEZ Hybridization System (ACD). Following the RNAscope assay, samples were counterstained for 2 minutes with 50% Gill's Hematoxylin diluted in dH_20. Hybridization signals were detected by sequential chromogenic reactions using red and green chromogens, and RNA staining signal was identified as red and green punctate dots. Each sample was quality controlled for RNA integrity with a probe specific to the *Ppib* housekeeping gene only samples with an average of >4 dots per cell were included for analysis. Negative control background staining was evaluated using a probe specific to the bacterial *dapB* gene; only samples with an average of <1 dot per 10 cells were included for analysis. *Cdkn1a* expression was quantitated using RNAscope SpotStudio (Wang et al., 2013). Outline regions of interest (ROI) were classified into individual dots and clustered dots within the ROI. Hematoxylin-stained nuclei and cell boundaries were also detected. RNAscope *Cdkn1a* mRNA images were batch analyzed after selecting appropriate ROIs manually. For each whole slide image, three ROIs were selected to represent the entire tumor section. The number of cells analyzed ranged from 4,000 to 43,000.

Pharmacokinetic analysis of MLN8237 and GDC-0449

Calibration and quality control solutions were prepared in dimethyl sulfoxide (DMSO). MLN8237 and GDC-0449 calibration standards used for spiking were prepared to give final matrix concentrations of 2-10000 nM. Quality Control (QC) standards were also prepared to give a final concentration of 25, 250, 750 and 2500 nM. A stock solution of Olomoucine (Sigma-Aldrich) internal standard (IS) was prepared in DMSO at a concentration of 1 mM and further diluted in methanol to give a working IS solution of 250 nM for quenching. Plasma and tissue samples were homogenized in either 3 or 5 ml/g phosphate buffered. 100 μl aliquots of untreated mouse plasma or tissue homogenates were spiked with 10 μl of the appropriate calibration or QC standard solutions. 100 μl aliquots of the unknown samples were spiked with 10 μl DMSO. Where necessary, plasma and tissue samples were diluted with untreated (blank) matrix. Spiked protein calibration standards, QCs and unknown samples were precipitated with 300 μL methanol containing 250 nM IS. Blank samples were prepared by spiking 100 μl untreated plasma/tissue with 10 μl DMSO and protein precipitated with 300 μl methanol. After centrifugation, supernatants were analyzed by liquid chromatography with tandem mass spectrometry detection (LC-MS/MS) using a Xevo TQ-S mass spectrometer coupled with an Acquity ultra-performance liquid chromatography UPLC H-class system (Waters). Chromatography was carried out using a Phenomenex C18 X-B column (2.6 μm, 50 mm x 2.1 mm ID) with a gradient mobile phase consisting of 0.1 % formic acid and methanol. 2ml of sample was injected on to the column using a flow rate of 0.6 ml/min with a 5 minute run time. Both analytes and IS were ionized using electrospray interface in positive ion mode. Detection was via tandem mass spectrometry (MS/MS) in multiple reaction monitoring (MRM) mode. The transitions m/z 519.12-139.04, 421.11-110.87 and m/z 299.19-177.29 were monitored for MLN8237, GDC-0449 and IS respectively. Data acquisition was performed using Targetlynx. The assay was linear over the range 2-10,000 nM.

Expression Microarray Analysis

Affymetrix HGU133plus2 expression profiles of primary tumors from 110 individuals with a diagnosis of medulloblastoma were taken from previously published studies (Fattet et al., 2009; Kool et al., 2008) (GSE12992 and GSE10327, respectively). Raw data were normalized and processed using gcRMA (Bioconductor/R). Processed data were converted to four metagenes representing the four sub-groups using non-negative matrix factorization (NMF) and these metagenes projected onto the mouse tumor/cell line expression profiles using an adaption of a previously described procedure (Tamayo et al., 2007) (scripts available upon request). Mouse expression profiles were generated using Illumina Mouse v8 arrays according to manufacturer's instructions. Raw data were processed using the beadarray package (Bioconductor/R). Expression profiles from primary murine medulloblastoma samples and cell lines included a total of 47 from GTML-based mouse models, 6 from a *Myc/Trp53*-deficient mouse model (Kawauchi et al., 2012) (gifted by Martine Roussel, St. Jude Children's Research Hospital) and 36 from *Ptch*$^{+/-}$ mice (Lastowska et al., 2013) (GSE43994). Also included were published *Ptch*$^{+/-}$ and *Myc/Trp53*-deficient mouse gene expression profiles (Kawauchi et al., 2012) (GSE34126 and GSE24628). Subgroup calls for mouse tumors were made using a support vector machine algorithm (SVM) trained on the four human subgroup metagenes and tested on the projected mouse metagenes.

SUPPLEMENTAL REFERENCES

Boland, C. R., Thibodeau, S. N., Hamilton, S. R., Sidransky, D., Eshleman, J. R., Burt, R. W., Meltzer, S. J., Rodriguez-Bigas, M. A., Fodde, R., Ranzani, G. N., and Srivastava, S. (1998). A National Cancer Institute Workshop on Microsatellite Instability for cancer detection and familial predisposition: development of international criteria for the determination of microsatellite instability in colorectal cancer. Cancer research *58*, 5248-5257.

Fattet, S., Haberler, C., Legoix, P., Varlet, P., Lellouch-Tubiana, A., Lair, S., Manie, E., Raquin, M. A., Bours, D., Carpentier, S.*, et al.* (2009). Beta-catenin status in paediatric medulloblastomas: correlation of immunohistochemical expression with mutational status, genetic profiles, and clinical characteristics. The Journal of pathology *218*, 86-94.

Grossmann, V., Kohlmann, A., Zenger, M., Schindela, S., Eder, C., Weissmann, S., Schnittger, S., Kern, W., Muller, M. C., Hochhaus, A.*, et al.* (2011). A deep-sequencing study of chronic myeloid leukemia patients in blast crisis (BC-CML) detects mutations in 76.9% of cases. Leukemia *25*, 557-560.

Jung, H. L., Wang, K. C., Kim, S. K., Sung, K. W., Koo, H. H., Shin, H. Y., Ahn, H. S., Shin, H. J., and Cho, B. K. (2004). Loss of heterozygosity analysis of chromosome 17p13.1-13.3 and its correlation with clinical outcome in medulloblastomas. J Neurooncol *67*, 41-46.

Kool, M., Jones, D. T., Jager, N., Northcott, P. A., Pugh, T. J., Hovestadt, V., Piro, R. M., Esparza, L. A., Markant, S. L., Remke, M.*, et al.* (2014). Genome Sequencing of SHH Medulloblastoma Predicts Genotype-Related Response to Smoothened Inhibition. Cancer cell *25*, 393-405.

Kool, M., Koster, J., Bunt, J., Hasselt, N. E., Lakeman, A., van Sluis, P., Troost, D., Meeteren, N. S., Caron, H. N., Cloos, J.*, et al.* (2008). Integrated genomics identifies five medulloblastoma subtypes with distinct genetic profiles, pathway signatures and clinicopathological features. PloS one *3*, e3088.

Lastowska, M., Al-Afghani, H., Al-Balool, H. H., Sheth, H., Mercer, E., Coxhead, J. M., Redfern, C. P., Peters, H., Burt, A. D., Santibanez-Koref, M.*, et al.* (2013). Identification of a neuronal transcription factor network involved in medulloblastoma development. Acta Neuropathol Commun *1*, 35.

Soussi, T., Asselain, B., Hamroun, D., Kato, S., Ishioka, C., Claustres, M., and Beroud, C. (2006). Meta-analysis of the p53 mutation database for mutant p53 biological activity reveals a methodologic bias in mutation detection. Clinical cancer research : an official journal of the American Association for Cancer Research *12*, 62-69.

Wang, F., Flanagan, J., Su, N., Wang, L. C., Bui, S., Nielson, A., Wu, X., Vo, H. T., Ma, X. J., and Luo, Y. (2012). RNAscope: a novel in situ RNA analysis platform for formalin-fixed, paraffin-embedded tissues. The Journal of molecular diagnostics : JMD *14*, 22-29.

Wang, Z., Portier, B. P., Gruver, A. M., Bui, S., Wang, H., Su, N., Vo, H. T., Ma, X. J., Luo, Y., Budd, G. T., and Tubbs, R. R. (2013). Automated quantitative RNA in situ hybridization for resolution of equivocal and heterogeneous ERBB2 (HER2) status in invasive breast carcinoma. The Journal of molecular diagnostics : JMD *15*, 210-219.
